# Inter-subject and inter-parcellation variability of resting-state whole-brain dynamical modeling

**DOI:** 10.1016/j.neuroimage.2021.118201

**Published:** 2021-08-01

**Authors:** Oleksandr V. Popovych, Kyesam Jung, Thanos Manos, Sandra Diaz-Pier, Felix Hoffstaedter, Jan Schreiber, B.T. Thomas Yeo, Simon B. Eickhoff

**Affiliations:** a Institute of Neuroscience and Medicine (INM-7), Research Centre Juelich, Juelich, Germany; b Institute of Systems Neuroscience, Medical Faculty, Heinrich-Heine, University Duesseldorf, Duesseldorf, Germany; c Laboratoire de Physique Théorique et Modélisation, CY Cergy Paris Université, CNRS, UMR 8089, Cergy-Pontoise cedex 95302, France; d Institute for Advanced Simulation, Juelich Supercomputing Centre (JSC), Research Centre Juelich, Juelich, Germany; e Institute of Neuroscience and Medicine (INM-1), Research Centre Juelich, Juelich, Germany; f Centre for Sleep and Cognition, Centre for Translational MR Research & N.1 Institute for Health, National University of Singapore, Singapore; g Department of Electrical and Computer Engineering, National University of Singapore, Singapore; h Martinos Center for Biomedical Imaging, Massachusetts General Hospital, Charlestown, MA, USA; i Integrative Sciences and Engineering Programme (ISEP), Singapore

**Keywords:** Brain atlas, Whole-brain model, Resting-state brain dynamics, Simulations, Brain connectome, Model validation

## Abstract

Modern approaches to investigate complex brain dynamics suggest to represent the brain as a functional network of brain regions defined by a brain atlas, while edges represent the structural or functional connectivity among them. This approach is also utilized for mathematical modeling of the resting-state brain dynamics, where the applied brain parcellation plays an essential role in deriving the model network and governing the modeling results. There is however no consensus and empirical evidence on how a given brain atlas affects the model outcome, and the choice of parcellation is still rather arbitrary. Accordingly, we explore the impact of brain parcellation on inter-subject and inter-parcellation variability of model fitting to empirical data. Our objective is to provide a comprehensive empirical evidence of potential influences of parcellation choice on resting-state whole-brain dynamical modeling. We show that brain atlases strongly influence the quality of model validation and propose several variables calculated from empirical data to account for the observed variability. A few classes of such data variables can be distinguished depending on their inter-subject and inter-parcellation explanatory power.

## 1 Introduction

Investigation of brain dynamics during task-evoked and resting-state activity is frequently based on the inspection of corresponding functional networks that are collections of brain regions with enhanced synchronization among them (Bolt, Nomi, Rubinov, Uddin, 2017, Cole, Bassett, Power, Braver, Petersen, 2014, Park, Friston, 2013). Neither nodes nor edges of such networks can uniquely be defined, especially, for the resting-state brain activity. State-of-the-art approaches range from voxel-wise nodes resulting in huge networks defined by the number of voxels in the underlying neuroimaging data to nodes encircling entire brain regions either as neuronal foci co-activated during a specific task or parcellated according to other criteria (Stanley et al., 2013). In the latter case, the brain regions are defined based on a certain brain parcellation (Eickhoff, Yeo, Genon, 2018, Stanley, Moussa, Paolini, Lyday, Burdette, Laurienti, 2013, Thirion, Varoquaux, Dohmatob, Poline, 2014), which reduces the dimensionality of the brain data by merging hundred thousands of voxels from high-resolution neuroimaging data into a few hundreds up to thousand of brain regions. A unified brain parcellation could improve the interpretability and comparability of results for different subjects and studies and increase the effective signal-to-noise ratio. However, there are many ways to parcellate the brain into separate regions (or parcels), which is actively debated in the literature (Eickhoff, Yeo, Genon, 2018, Stanley, Moussa, Paolini, Lyday, Burdette, Laurienti, 2013, Thirion, Varoquaux, Dohmatob, Poline, 2014). There is a sparse empirical evidence for the effect of a particular atlas choice, but see Refs. (Messe, 2019, Pervaiz, Vidaurre, Woolrich, Smith, 2020, Zimmermann, Griffiths, Schirner, Ritter, McIntosh, 2019) for recent reports.

The great variety of possible techniques for creating brain parcellations and existing brain atlases makes the choice of a particular parcellation for a given analysis very difficult (Eickhoff et al., 2018a). At least two paradigmatically distinct approaches can be used for the parcellation, where the brain regions are defined based either on their anatomical or functional properties. For example, the cortex can be parcellated into regions according to its folding properties, e.g., into gyral-based parcels encircled by tracing from the depth of one sulcus to another (Desikan et al., 2006). A very different parcellation approach is based on the brain function, where the patterns of the resting-state functional connectivity (FC) can be used to group the voxels (or vertices) into parcels of similar connectivity (Schaefer, Kong, Gordon, Laumann, Zuo, Holmes, Eickhoff, Thomas, 2018, Shen, Tokoglu, Papademetris, Constable, 2013). The latter can be evaluated either according to a global similarity measure combined with abrupt changes in the local gradient of the whole-brain intrinsic FC (Schaefer et al., 2018) or based on the graph theory with application of a multigraph clustering approach to the resting-state FC (Shen et al., 2013). From the above anatomical and functional approaches to brain parcellation, one may assume that the latter could be more appropriate for calculation of the whole-brain FC, where the parcels are suspected to be composed of voxels with higher functional homogeneity. However, the detailed effects of these two distinct parcellation techniques on the results of data analysis and modeling can hardly be predicted by a simple theoretical reasoning.

Utilizing a brain parcellation is essential for dynamical modeling of brain activity, where the brain regions are represented as nodes of a network model (Honey et al., 2009). The selected brain parcellation is involved in the extraction of the structural connectivity (SC), inferred from diffusion-weighted magnetic resonance imaging (dwMRI), which serves as proxies for anatomical connections between brain regions at the meso- and macroscopic level (Hagmann et al., 2010). This SC can then be used to estimate the coupling strength and communication delay between the nodes of the model network contributing in such a way to the model derivation (Deco, Jirsa, McIntosh, 2011, Ghosh, Rho, McIntosh, Kotter, Jirsa, 2008). Furthermore, the selected parcellation can be used to extract the blood oxygen level-dependent (BOLD) signals inferred from functional magnetic resonance imaging (fMRI) and calculate the empirical FC. The latter can be compared to simulated FC calculated from simulated BOLD time series generated by the derived model, thus validating the simulation results against the empirical data (Cabral, Hugues, Sporns, Deco, 2011, Deco, Jirsa, 2012). As a consequence, this process crucially depends on the empirical data used for the model derivation (e.g., SC) and fitting (e.g., FC), which in turn is affected by the data processing, in particular, by the selected brain parcellation (Messe, 2019, Pervaiz, Vidaurre, Woolrich, Smith, 2020, Popovych, Manos, Hoffstaedter, Eickhoff, 2019, Zimmermann, Griffiths, Schirner, Ritter, McIntosh, 2019).

In this study we therefore simulate the resting-state brain activity using dynamical mathematical models to investigate the effects of brain parcellations. Functional and anatomical brain atlases with different resolutions are used for model validation against empirical resting-state functional and structural connectivity data. We consider three representatives from the above parcellation classes as given by the anatomical Harvard-Oxford atlas (Desikan et al., 2006) and the functional Schaefer (Schaefer et al., 2018) and Shen (Shen et al., 2013) atlases. The effects of brain parcellation are studied in detail with two systems of coupled phase and limit-cycle oscillators suggested for modeling cortical oscillations and resting-state BOLD dynamics (Breakspear, Heitmann, Daffertshofer, 2010, Cabral, Hugues, Sporns, Deco, 2011, Deco, Cruzat, Cabral, Tagliazucchi, Laufs, Logothetis, Kringelbach, 2019, Deco, Kringelbach, Jirsa, Ritter, 2017, Fukushima, Sporns, 2018, Ponce-Alvarez, Deco, Hagmann, Romani, Mantini, Corbetta, 2015). The effects are investigated by an extensive exploration of the model parameter space. The models are fitted against empirical data of individual subjects for a set of varying conditions, in particular, the granularity of the parcellation for Schaefer and Shen atlases and the maximal probability threshold for Harvard-Oxford atlas affecting the size of brain regions.

The number of parcels is an important parameter, which may influence the results of the mathematical modeling, the empirical structure-function relationship as well as the prediction of human behavior from the patterns of brain connectivity (Honey, Sporns, Cammoun, Gigandet, Thiran, Meuli, Hagmann, 2009, Messe, 2019, Pervaiz, Vidaurre, Woolrich, Smith, 2020, Proix, Spiegler, Schirner, Rothmeier, Ritter, Jirsa, 2016, Zimmermann, Griffiths, Schirner, Ritter, McIntosh, 2019) and deserves a systematic modeling investigation (Popovych et al., 2019). In the paper (Proix et al., 2016) the authors explored the impact of parcellations and local connectivity on the dynamics of neural mass models with and without delays, where the different parcellations were obtained by randomly splitting the brain regions of the Desikan-Killiany atlas into smaller subregions. It was in particular identified that spatial attractors of slow brain dynamics were qualitatively not affected by the number of regions in the cortical parcellation, whereas the parcellation granularity influenced their critical range in the global coupling strength. On the other hand, the richness of fast dynamics of the response to perturbations increased only if delays were considered in the model, suggesting an optimal parcellation scale, which can be decomposed into only a few spatial patterns. The work of Zimmermann et al. (2019) exposed a subject specificity to the association between empirical structural and functional connectomes for six different datasets and brain parcellations. It was however shown that intra-subject specificity of the SC-FC fit was achieved only for one of the considered cases indicating that selecting an appropriate brain parcellation was critical to provide enough statistical information to individually link SC and FC. The structure-function relationships between empirical SC and FC were also investigated for several brain parcellations with various spatial resolutions by Messe (2019) revealing a significant effect of brain parcellation on the SC-FC correlation driven by the number of brain regions. In the paper (Pervaiz et al., 2020) the impact of brain parcellation on the predictive power of data-driven models was analyzed regarding the relationship between whole brain functional connectivity patterns and behavioral traits in an attempt to find the optimal parcellation among other conditions.

In this study we analyze the parcellation-induced differences of model validation against empirical data for two approaches to brain parcellation based on anatomical or functional brain data. Furthermore, we test for an effect on two different models of limit-cycle and phase oscillators distinguished whether the amplitude of the simulated BOLD signals is taken into account or not, respectively. We consider functional and structure-functional fitting modalities for the model validation against empirical data. We aim to evaluate whether and how different parcellations may influence the modeling results and suggest possible approaches to explain inter-subject and inter-parcellation variation of model fitting. In our approach, we study the contribution of different features of the experimental data, which can vary with the pre-processing and chosen parcellation, to the ability of mathematical models to make an individualized link between simulated and empirical connectomes. We demonstrate that the considered atlases lead to substantially different results when comparing the model fit for parcellations within and between the anatomical and functional parcellation families. This is especially the case for the quality of the model validation, structure of the model parameter space and reliability of the fitting results. To understand the origin of the observed behavior of the model fitting, we also evaluate how the properties of the empirical data used for model derivation and validation may influence the modeling results (Messe et al., 2014). We show that several data variables calculated from the empirical neuroimaging data can be classified into a few correlative types depending on their contribution to the model fitting for individual subjects and for the brain parcellations from the same or different brain atlases. In this respect, the variation of the fitting results for personalized models across subjects and parcellations can, to a greater extent, be accounted for by the variation of the considered data variables.

## 2 Methods and materials

### 2.1 Empirical data

Empirical SC and FC used for the derivation and validation of the mathematical models were extracted for 272 healthy unrelated subjects (144 females, average age 28.5 ± 3.5 [mean±std] years) from the Human Connectome Project (HCP; https://www.humanconnectome.org/) (Van Essen et al., 2013) S1200 public release with complete dwMRI and resting-state fMRI data.    

*Structural connectivity* Empirical SC approximating the anatomical axonal tracts in the brain (Conturo et al., 1999) was extracted from pre-processed dwMRI data. To do this, we developed an in-house pipeline consisting of FSL version 5.0 (Jenkinson et al., 2012), Freesurfer 6.0 (Fischl et al., 2001), ANTs 3.0 (Tustison et al., 2014), and MRtrix3 3.0 (Tournier et al., 2019). The main pre-processing steps included de-noising, bias-field correction, removal of eddy-current-induced distortions and motion correction (dwMRI), normalization of image intensity (T1-weighted image), co-registering the diffusion data with the T1-weighted image, estimation of the transformation function from the MNI standard template to the native diffusion space, and segmentation and application of tissue masks in the diffusion space. Then the whole-brain tractography (WBT) was calculated by the probabilistic fiber tracking algorithm (iFOD2) based on the multi-shell-multi-tissue constrained spherical deconvolution algorithm (Jeurissen et al., 2014), which was realized in MRtrix3, where 10 million streamlines were obtained. The tracking algorithm used voxels in the white-mater mask for seeding of tracts with the maximal angle in 45 degrees between successive steps. Finally, the resulting SC was extracted from the calculated WBT according to a given brain parcellation defining a set of brain regions (parcels), where any two parcels were selected as seed and target regions for the compression of WBT to the parcellation-based SC. The output is two N×N matrices of SC containing the empirical streamline counts (eSC) and the averaged empirical streamline path lengths (ePL) between any pair from N brain regions of the considered brain parcellation.    

*Resting-state functional connectivity* The empirical FC was calculated from the resting-state fMRI data which was ICA FIX denoised as provided by the HCP repository (Glasser, Sotiropoulos, Wilson, Coalson, Fischl, Andersson, Xu, Jbabdi, Webster, Polimeni, Van Essen, Jenkinson, 2013, Griffanti, Salimi-Khorshidi, Beckmann, Auerbach, Douaud, Sexton, Zsoldos, Ebmeier, Filippini, Mackay, Moeller, Xu, Yacoub, Baselli, Ugurbil, Miller, Smith, 2014, Salimi-Khorshidi, Douaud, Beckmann, Glasser, Griffanti, Smith, 2014). Similar to the extraction of the empirical SC, also for the calculation of the empirical FC, the brain was split into a set of regions according to a given brain parcellation, and the mean BOLD signals (averaged over all voxels in any region) were calculated for all parcels. The extracted BOLD signals were then cross-correlated by Pearson correlation resulting in N×N empirical FC (eFC) matrices for each subject. The HCP repository provided 4 resting-state fMRI sessions (1200 volumes, TR = 720 ms) for each considered subject corresponding to the scans with two different phase-encoding directions repeated on two different days. This accordingly resulted in 4 eFC matrices for each subject. Additionally, the BOLD signals from all 4 scanning sessions were concatenated, and 5 eFC matrices were obtained in total for each subject.    

*Brain parcellation* The empirical SC and FC were calculated for 11 brain parcellations using the Schaefer and Shen atlases based on the resting-state functional connectivity (Schaefer, Kong, Gordon, Laumann, Zuo, Holmes, Eickhoff, Thomas, 2018, Shen, Tokoglu, Papademetris, Constable, 2013), and the Harvard-Oxford atlas based on the anatomy of cortical folding (Desikan et al., 2006). Several variations of these atlases were considered: the Schaefer atlas with 100, 200, 400 and 600 cortical parcels (denoted as S100, S200, S400 and S600, respectively), the Shen atlas with 79, 156 and 232 cortical regions (denoted as Shen79, Shen156 and Shen232), and the probabilistic Harvard-Oxford atlas with 96 non-overlapping cortical parcels with thresholds at 0%, 25%, 35%, and 45% of the maximal probability (denoted as HO96 0%, HO96 25%, HO96 35%, and HO96 45%, respectively). For higher thresholding, voxels that did not reach the threshold level were excluded, and for 45% threshold the left supracalcarine cortex region contained no supra threshold voxels reducing the number of parcels to 95 for HO96 45%.

**Fig. 1:**
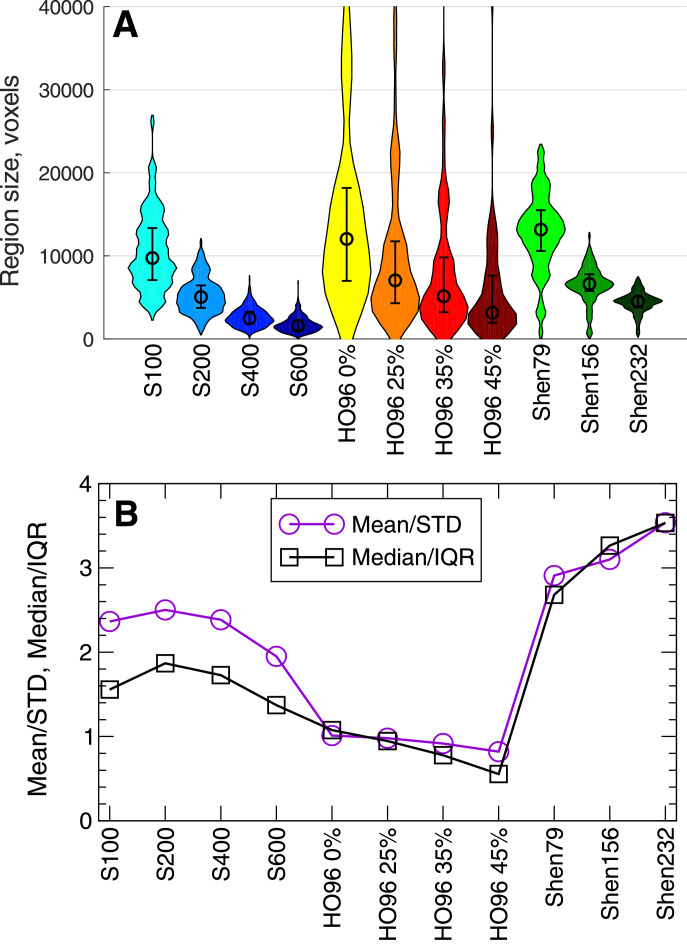
Variation of the region size for the considered brain parcellations. (**A**) Distributions of the region size (the number of 1 mm isocubic voxels) and (**B**) the corresponding relations between the mean or median and the spread of the region size are depicted versus all considered parcellations. The spread of the region size is reflected by the standard deviation (STD) or interquartile range (IQR) as indicated in the legends.

Finer granularity for the Schaefer and Shen atlases and larger threshold for the Harvard-Oxford atlas led to smaller brain regions of the corresponding parcellations as illustrated in Fig. 1A. The main difference between the considered atlases is that the brain regions are more homogeneous in size for the Schaefer and Shen atlases than for the Harvard-Oxford atlas. However, the size spread decayed together with the average size such that the relations between them little changed for varying granularity and probability threshold, albeit overall differences between the three parcellation families [Fig. 1B]. The variation of the atlases, their parcellation granularity and probability threshold affected the properties of the empirical data used for the model derivation and validation as discussed in Section 3.3 below.

### 2.2 Models and simulated data

In this study we considered two models. The first model is an ensemble of coupled phase oscillators of Kuramoto type (Kuramoto, 1984)

(1) $$\begin{array}{c} \;{\dot{\varphi }}_{j}\left(t\right)=2\pi f_{j}+\frac{C}{N}\sum_{n=1}^{N}w_{jn}\sin\left(\varphi_{n}\left(t-\tau_{jn}\right)-\varphi_{j}\left(t\right)\right)+\eta_{j},\\ j=1,2,\ldots ,N,\; \end{array}$$

where $\varphi_{j}$ are the phases, N is the number of oscillators, $f_{j}$ are the natural frequencies (frequencies of the uncoupled oscillators, measured in hertz (Hz), and the time t in the model and delay in coupling are thus measured in seconds), and C is the parameter of the global coupling. Parameters $w_{jn}$ and $\tau_{jn}$ represent the individual coupling weight and propagation delay in the coupling, respectively, from oscillator n to oscillator j, and $\eta_{j}$ is an independent noise uniformly distributed in the interval [−0.3,0.3]. This system was used to model by the observable $x_{j}=\sin\left(\varphi_{j}\right)$ the dynamics of the empirical BOLD signal of the jth brain region (parcel) according to a given brain parcellation as explained above, where the number of oscillators N in model (1) was equal to the number of brain parcels.

Another investigated model is a system of coupled generic limit-cycle (LC) oscillators that are the normal form of the supercritical Hopf bifurcation (Kuznetsov, 1998)

(2) $$\begin{array}{ccc} {\dot{z}}_{j}\left(t\right) & = & \left(a_{j}+i2\pi f_{j}-|z_{j}\left(t\right){|}^{2}\right)z_{j}\left(t\right)\\ & & +\frac{C}{N}\sum_{n=1}^{N}w_{\mathrm{jn}}\left(z_{n}\left(t-\tau_{\mathrm{jn}}\right)-z_{j}\left(t\right)\right)+\xi_{j},\\ & & \;j=1,2,\cdots ,N, \end{array}$$

where $z_{j}\left(t\right)=x_{j}\left(t\right)+iy_{j}\left(t\right)$ are the complex variables of individual oscillators, and $i=\sqrt{-1}$ is the imaginary unit. Without coupling (C=0), all oscillators of ensemble (2) independently and uniformly rotate around the origin on the limit cycles with individual radii $\sqrt{a_{j}}$ and with individual natural frequencies $f_{j}$ measured in Hz. The independent complex noise $\xi_{j}$ is uniformly distributed in the interval [−0.3,0.3]. The empirical BOLD signal of region j was modeled by the variable $x_{j}\left(t\right)$.

The model parameters $f_{j}$, $a_{j}$, $w_{jn}$ and $\tau_{jn}$ are extracted from the empirical data for each individual subject, and the personalized models (1) and (2) were simulated separately for each subject. The natural frequencies $f_{j}$ of the phase and LC oscillators were calculated from the empirical BOLD signals extracted from the corresponding brain regions as the frequencies of the maximal spectral peaks discarding the frequencies below 0.01 Hz and above 0.1 Hz. Similar approach for defining the local model parameters was also used in other studies for the phase and LC oscillators (Deco, Cruzat, Cabral, Tagliazucchi, Laufs, Logothetis, Kringelbach, 2019, Deco, Kringelbach, Jirsa, Ritter, 2017, Ponce-Alvarez, Deco, Hagmann, Romani, Mantini, Corbetta, 2015). The amplitude parameters $a_{j}$ of LC oscillators (2) were selected proportionally to the extent of time fluctuations of empirical BOLD signals of individual parcels. For this, the normalized standard deviation $std({\text{BOLD}}_{j})$ was used to calculate $a_{j}$ such that the mean and the standard deviation over all parcels were $\langle a_{j}\rangle =0.5$ and $std(a_{j})=0.4$, respectively.

The coupling weights $w_{jn}$ and delays $\tau_{jn}$ were derived from the eSC and ePL, respectively. The parameters $w_{jn}$ were calculated as the normalized number of SC streamlines $w_{jn}=k_{jn}/\langle k_{jn}\rangle$, where $k_{jn}$ is the number of streamlines connecting regions j and n, and 〈·〉 denotes the ensemble averaging over the entire N×N matrix with zero diagonal. The matrix of the streamline counts $eSC=\left\{k_{jn}\right\}$ thus defined the coupling weights and the graph of the model network. The delays $\tau_{jn}$ were calculated as $\tau_{jn}=L_{jn}/V$, where $L_{jn}$ is the average path length of the streamlines connecting regions j and n, and V is an average velocity of signal propagation. The matrix $ePL=\left\{L_{jn}\right\}$ can thus be used to calculate the delays $\tau_{jn}$ in the coupling, which can be rewritten as $\tau_{jn}=\tau \cdot L_{jn}/\langle L_{jn}\rangle$, where $\tau =\langle L_{jn}\rangle /V$ is the global (or average) delay. In models (1) and (2) the self-connections were excluded ($w_{jj}=0$) by putting the diagonal elements in the matrices eSC and ePL to zero: $k_{jj}=L_{jj}=0$. The parameters of the global coupling C and the global delay τ can be used to scale the extent of the coupling in the system and the average velocity V, respectively, and were varied to fit the model to empirical data.

#### 2.2.1 Model validation

**Fig. 2:**
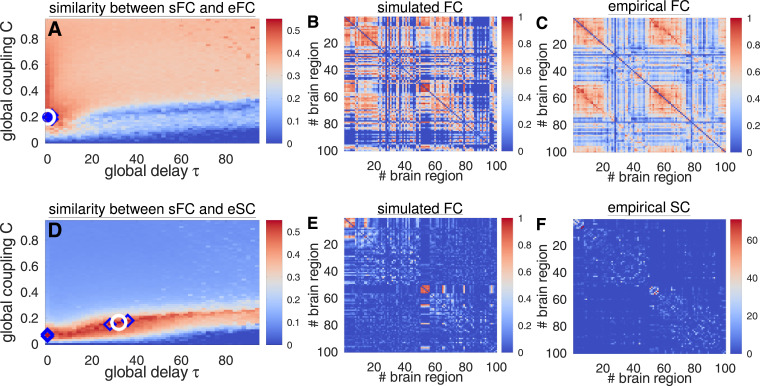
Examples of model (1) validation against empirical data. Fitting of the simulated FC (sFC) to eFC (upper row, **A-C**) and to eSC (lower row, **D-F**) for S100 parcellation. (**A, D**) Similarity (Pearson correlation coefficient) between the simulated and empirical data is encoded in color versus parameters of the global delay τ and coupling C, where the optimal parameter points of the best fit are indicated by white circles, and the next 4 largest values are depicted by blue diamonds. The corresponding sFC matrices of the best fit compared with eFC and eSC, respectively, are depicted in the middle column (**B** and **E**), whereas the corresponding eFC matrix and normalized by its mean eSC matrix are shown in the right column of the upper (**C**) and lower (**F**) row, respectively. The simulated and empirical FC matrices are shown in the same scale for comparison. (For interpretation of the references to colour in this figure legend, the reader is referred to the web version of this article.)

For each set of the model parameters, the models (1) and (2) were numerically simulated, and the matrix of the simulated functional connectivity (sFC) was calculated by Pearson correlation between the simulated BOLD signals $x_{j},\;j=1,2,\cdots ,N$. sFC was compared with the matrices of the empirical connectivity eFC and eSC, where the similarity between them was calculated by Pearson correlation, i.e., corr(sFC,eFC) or corr(sFC,eSC) between the corresponding upper triangular parts. The model fitting for the phase oscillators (1) is illustrated in Fig. 2. For given eFC and eSC [Fig. 2C and F], the model parameters τ and C were varied, and the similarity between sFC and the empirical connectivity matrices was calculated for each parameter point (τ,C) [Fig. 2A and D]. Among all tested parameter values, the optimal values were selected corresponding to the best model fit, i.e., where the similarity is maximal [Fig. 2A and D, while circles]:

(3) $$\begin{array}{c} \text{Fit}\left(\text{sFC},\text{eFC}\right)=\underset{\left(C,\;\tau \right)}{\max\;\;\;}\text{corr}\left(\text{sFC},\text{eFC}\right),\\ \text{Fit}\left(\text{sFC},\text{eSC}\right)=\underset{\left(C,\;\tau \right)}{\max\;\;\;}\text{corr}\left(\text{sFC},\text{eSC}\right). \end{array}$$

The goodness-of-fit values Fit(sFC,eFC) of the functional model fitting can be used to evaluate the similarity between the simulated patterns of synchronization between oscillators of systems (1) and (2) and the resting-state BOLD dynamics as given by eFC matrix. On the other hand, the structure-functional model fitting Fit(sFC,eSC) evaluates how strongly the model dynamics can replicate the underlying network structure as for the structure-function relationship in the brain (Honey, Sporns, Cammoun, Gigandet, Thiran, Meuli, Hagmann, 2009, Messe, 2019, Park, Friston, 2013, Zimmermann, Griffiths, Schirner, Ritter, McIntosh, 2019) and for which parameters and dynamical regimes. Examples of the correspondence between sFC and empirical data are illustrated in Fig. 2, compare sFC matrices in Fig. 2B and E with eFC and eSC in Fig. 2C and F, respectively. For further analysis, optimal model parameters were selected from each parameter space as in Fig. 2A and D (white circles) together with the corresponding maximal similarity values Fit(·,·), i.e., goodness-of-fit of the model defined by Eq. (3).

As mentioned above, the two models were simulated for 11 brain parcellations (4 for the Schaefer atlas, 4 for the Harvard-Oxford atlas and 3 for the Shen atlas) defining 11 simulation conditions for each subject. Simulation for each condition resulted in 5 parameter planes like in Fig. 2A and D of comparison between sFC and eFC (each subject had 5 eFCs), and one plane of comparison between sFC and eSC. Each parameter plane spanned the range [0,94]×[0,0.945] of the coupling delay τ and strength C, respectively, and contained a grid of 48×64 parameter points. For each of these parameter points the models were numerically simulated (model run) for random initial conditions by the stochastic Heun integration method with fixed Δt=0.06 s integration step during 4000 s, where the last 3500 s were used for sFC evaluation (the first 500 s were skipped as transient). From each parameter plane one optimal parameter point (τ,C) was extracted and collected for further analysis [Fig. 2A and D, white circles], where the maximal similarities (3) were reached. For the considered 272 subjects we analyzed 272 × 5 = 1360 maximal similarities ${\text{Fit}}_{i}\left(\text{sFC},\text{eFC}\right)$ (i=1,2,⋯,1360) and 272 values of ${\text{Fit}}_{i}\left(\text{sFC},\text{eSC}\right)$ (i=1,2,⋯,272) and the corresponding optimal parameters $\left(\tau_{i},C_{i}\right)$ for each of 11 simulation conditions (brain parcellations) and 2 models. These values were derived from more than 18 millions of model runs.

For statistical analyses, we related the vectors ${\text{Fit}}_{i}\left(\cdot ,\cdot \right)$ (we omit the subscripts in what follows) across subjects between different brain parcellations and models to evaluate the similarity and interdependencies between modeling results with regard to simulation conditions (parcellations and models) as well as statistical properties of the empirical data. The similarity was evaluated by the Pearson correlation coefficients and their statistical significance as provided by the *corrcoeff* function in Octave. Fischers z-transform was applied to the correlation coefficients before (and after) performing arithmetic operations (e.g., averaging) and testing. For multivariate analysis the standard multiple linear regression model (MLR) was employed.

## 3 Results

In what follows we first illustrate the results of the model fitting for all considered subjects, parcellations, fitting modalities and models. Then we present two approaches to evaluate and explain the impact of brain parcellations on the inter-subject and inter-parcellation variability of the obtained modeling results. As our first approach, the results of the model fitting, i.e., the Fit-values of the maximal similarity (3) and the corresponding optimal model parameters (τ,C) were compared across individual subjects and between different brain parcellations and models. We evaluated the inter-parcellation variability of the fitting patterns across individual subjects. In the second approach, several data variables were calculated from individual empirical data and used to account for the variation of the goodness-of-fit across subjects for each of the considered brain parcellations as well as among them. Thereby, we assess the influence of individual data properties on intra- and inter-parcellation variability of the model fitting.

### 3.1 Results of model fitting

**Fig. 3:**
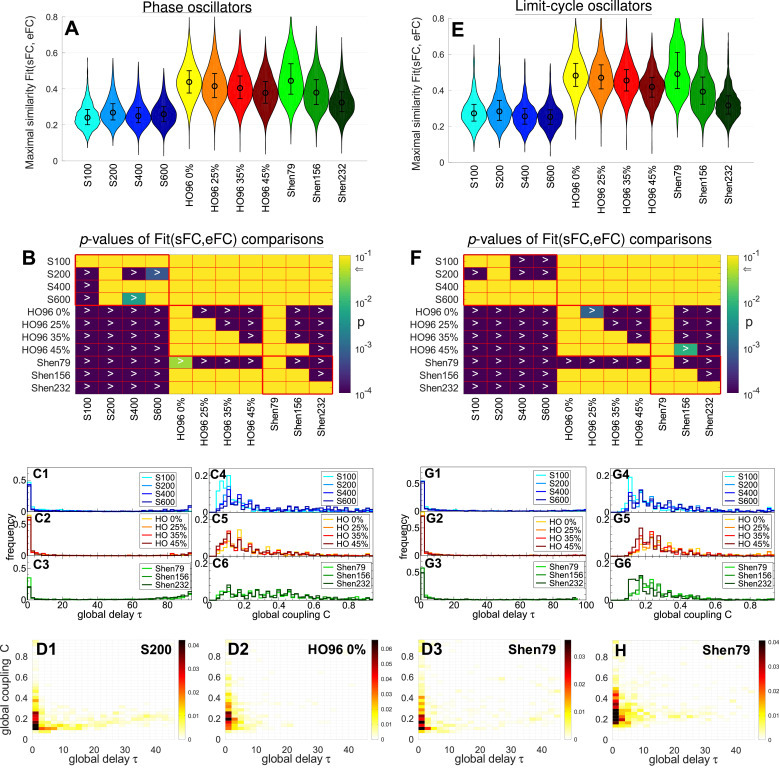
Results of the functional model fitting for (**A - D**) phase model (1) and (**E - H**) LC model (2). (**A, E**) Distributions of the maximal similarity values Fit(sFC, eFC) as violin plots for the considered brain parcellations denoted on the horizontal axes as introduced in Methods, where the medians and the interquartile ranges are also shown. (**B, F**) Outcomes of statistical tests, where the p-values (corrected for multiple comparisons) of the paired Wilcoxon signed-rank test of the Fit(sFC, eFC) values between the parcellations indicated on the axes are depicted by color in logarithmic scale (see color bar). The null hypothesis is rejected with p<.05 (indicated by arrow on the color bar) in favor of the alternative hypothesis ${\text{Fit}}^{\left(\text{row}\right)}>\;{\text{Fit}}^{\left(\text{column}\right)}$ for parcellations in the row and column, respectively, where the corresponding cell is dark (small p-value) and contains the inequality sign “>”. (**C,D,G,H**) Distributions of the corresponding optimal model parameters, where the one- and two-dimensional histograms of the occurrence frequency of the optimal parameters are, respectively, plotted as step-wise curves (**C, G**) and depicted in color (**D, H**) ranging from white (small values) to black (large values) for the parcellations indicated in the legends and plots. (For interpretation of the references to colour in this figure legend, the reader is referred to the web version of this article.)

The distributions of the maximal similarity Fit(sFC, eFC) of the fitting sFC to eFC are illustrated in Fig. 3A and E for the considered brain atlases and the two simulation models. The impact of the atlases is apparent when comparing the differences between Fit(sFC, eFC) for the Schaefer atlas (S100-S600, blue violins), the Harvard-Oxford atlas (HO96 0%-45%, yellow - dark red violins) and the Shen atlas (Shen79-Shen232, green violins). In the latter cases (HO96 and Shen) the both models demonstrate much higher fitting to the empirical data with up to 80% of the relative increase of Fit(sFC, eFC) with respect to S100-S600 cases [supplementary Table A.1]. The differences in the model fitting can also be observed between the parcellations of the same type, i.e., from the same atlas. In particular, the best fit for the Schaefer atlas was obtained for S200 case providing an optimal spatial scale for this brain atlas. For other atlases Fit(sFC, eFC) monotonically decays when the threshold for HO96 atlas or the number of parcels for the Shen atlas increases [Fig. 3A and E].

Results of a systematic statistical testing of Fit(sFC, eFC) for all considered simulation conditions (11 parcellations) are illustrated in Fig. 3B and F, where the p-values of the paired Wilcoxon signed-rank test are depicted in color for comparisons between different parcellations. The dark color (darker than yellow) at the intersection of a particular row and column of the shown matrices indicates that the goodness-of-fit for the condition from the vertical axis ${\text{Fit}}^{\left(\text{row}\right)}$ is statistically larger (with p<.05 at least) than ${\text{Fit}}^{\left(\text{column}\right)}$ for the condition from the horizontal axis accordingly. For example, ${\text{Fit}}^{\left(S200\right)}>{\text{Fit}}^{\left(S100\right)}$ as well as ${\text{Fit}}^{\left(S200\right)}>{\text{Fit}}^{\left(S400\right)}$ and ${\text{Fit}}^{\left(S200\right)}>{\text{Fit}}^{\left(S600\right)}$, where the cells at the intersection of the row “S200” and columns “S100”, “S400” and “S600” are dark and marked by “>” implying p<.05. We also confirm that the quality of the model fitting decays for larger probability threshold for HO96 atlas and for more parcels for the Shen atlas [Fig. 3B and F]. Shen79 provides the best fit for both models, whereas the lowest goodness-of-fit was obtained for S100 for the phase model and for S400 and S600 for the LC model, see the row “Shen79” and columns “S100”, “S400” and “S600” in Fig. 3B and F. The effect size associated with the presented p-values is illustrated in supplementary Fig. A.1.

The maximal similarity Fit(sFC, eFC) is achieved at the optimal model parameters as illustrated in Fig. 2A (white circle). Distributions of the optimal model parameters (τ,C) for the model fitting to the empirical functional data eFC for all subjects are shown as one-dimensional histograms in Fig. 3C and G, and as two-dimensional histograms in Fig. 3D and H for a few selected parcellations. We found that Fit(sFC, eFC) is attained at the optimal parameters remarkably concentrated towards small delay τ and moderate values of coupling C for all considered brain parcellations and models. Somewhat broader distribution of the optimal coupling can be observed for the Shen atlas for the phase model but not for the LC model [Fig. 3C6 and G6]. Further examples of the parameter planes averaged over all subjects are illustrated in supplementary Fig. A.2 together with the distributions of the optimal model parameters taking into account up to 5 largest similarity values per individual parameter plane [Fig. 2A and D, white circles and blue diamonds].

**Fig. 4:**
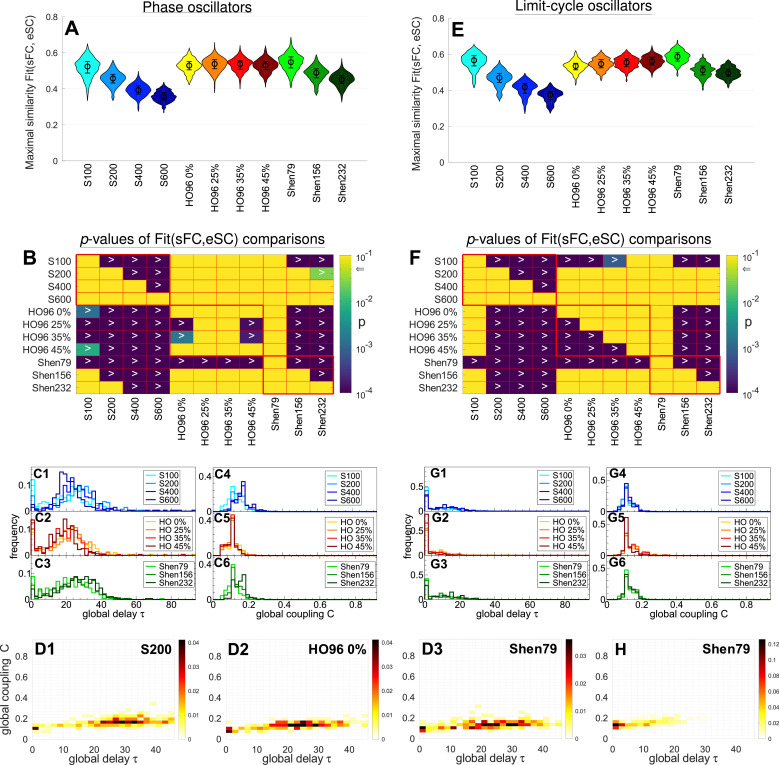
Results of the structure-functional model fitting for (**A - D**) phase model (1) and (**E - H**) LC model (2). (**A, E**) Distributions of the maximal similarity values Fit(sFC, eSC) for the considered brain parcellations, where the medians and the interquartile ranges are also shown. (**B, F**) Outcomes of statistical tests, where the corrected for multiple comparisons p-values of the paired Wilcoxon signed-rank test of the Fit(sFC, eSC) values between the parcellations indicated on the axes are depicted by color in logarithmic scale (see color bar). The null hypothesis is rejected with p<.05 (indicated by arrow on the color bar) in favor of the alternative hypothesis ${\text{Fit}}^{\left(\text{row}\right)}>\;{\text{Fit}}^{\left(\text{column}\right)}$ for parcellations in the row and column, respectively, where the corresponding cell is dark (small p-value) containing the inequality sign “>”. (**C,D,G,H**) Distributions of the corresponding optimal model parameters, where the one- and two-dimensional histograms of the occurrence frequency are, respectively, plotted as step-wise curves (**C, G**) and depicted in color (**D, H**) ranging from white (small values) to black (large values) for the parcellations indicated in the legends and plots. (For interpretation of the references to colour in this figure legend, the reader is referred to the web version of this article.)

The situation is different for the structure-function relationship, where sFC is fitted to eSC (count matrix) [Fig. 2D-F] as illustrated in Fig. 4. In particular, the maximal similarity monotonically decays in a well-pronounced manner when the granularity of the Schaefer and Shen atlases decreases for both models [Fig. 4A and E, blue and green violins, supplementary Table A.1 ]. In contrast, Fit(sFC, eSC) increases for larger threshold for HO96 atlas and the LC model [Fig. 4E and F, yellow-red violins]. On the other hand, the behavior of the Fit-values is non-monotonic for the phase model, where the thresholds of 25% and 35% are optimal for the structure-functional model fitting for HO96 atlas and phase model [Fig. 4A and B]. The highest and the lowest correspondence between the simulated and empirical data was obtained for Shen79 and S600, respectively, for both models, see also supplementary Fig. A.1 for effect size.

The distributions of the optimal model parameters for Fit(sFC, eSC) also exhibit a deviation from those for Fit(sFC, eFC) as illustrated in Fig. 4 (compare to Fig. 3). Interestingly, the best structure-functional model fitting can be achieved for small and very well localized values of the global coupling C and for broadly distributed delay τ [Fig. 4C and D] when compared to the functional fitting modality. The latter property is somewhat reduced for the LC model as compared to the phase model [Fig. 4G and H]. Nevertheless, positive delay in coupling is still important to obtain the best model fitting in this case for both models, see supplementary Fig. A.2 for more details and comparison between the phase and LC models.

### 3.2 Inter-parcellation variability of fitting results

To explore the variability of the fitting results over brain parcellation, in this section we analyze the similarity among the goodness-of-fit vectors Fit(·,·) (3) collected for all subjects and fMRI scan sessions (see Methods) calculated for different parcellations and models. The Fit-values were correlated across subjects for any two parcellations for the same as well as different models to evaluate how strongly the variation of the brain parcellation and model can affect the inter-subject patterns of the goodness-of-fit and assess the reliability of the fitting results.

**Fig. 5:**
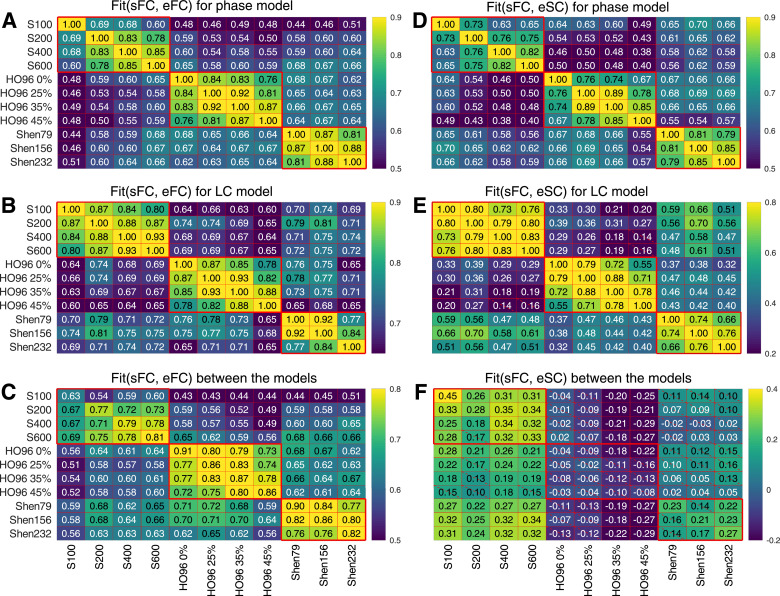
Correspondence between the patterns of the inter-individual variation of the fitting results (Fit-values (3)) for the considered parcellations and models. The vectors of the Fit-values collected over all subjects and scans (see Methods for details) were Pearson correlated with each other for any two parcellations (indicated on the axes) for (**A - C**) Fit(sFC, eFC) and (**D - F**) Fit(sFC, eSC), and for (**A, D**) phase model and (**B, E**) LC model. In plots (**C** and **F**) the correspondence between the phase model (parcellations on the vertical axes) and LC model (parcellations on the horizontal axes) is illustrated. The results are depicted by color, and their magnitudes are indicated in the plots. The crossed out cells indicate that the corresponding correlation does not reach the statistical significance with p<.05. The heavy red lines delineate the parcellations from the same atlas (parcellation family). (For interpretation of the references to colour in this figure legend, the reader is referred to the web version of this article.)

The pairwise correlations of the maximal similarity Fit(sFC, eFC) between any two of the considered brain parcellations are shown for the phase model in Fig. 5A and LC model in Fig. 5B. We observe that the fitting results are well correlated for parcellations within the same atlas/parcellation family, i.e., among S100-S600 parcellations and within HO96 and Shen atlases. The average intra-atlas correlations are 0.82 for the phase model [Fig. 5A] and 0.86 for the LC model [Fig. 5B]. On the other hand, the similarity of the model fitting patterns between different atlases is reduced, which holds for both models, and the corresponding average inter-atlas correlations are 0.59 and 0.71, for the phase and LC models, respectively. The inter-subject patterns of the goodness-of-fit Fit(sFC, eFC) are preserved for both dynamical models as illustrated in Fig. 5C, where the phase model was used for parcellations on the vertical axis, and the LC model was simulated for parcellations on the horizontal axis. As for the inter-parcellation correspondence of the fitting results for the same model [Fig. 5A and B], similar amount of stronger intra- and weaker inter-atlas correlation is observed for the between-model comparison [Fig. 5C].

The same conclusion can be drawn for the structure-functional model fitting Fit(sFC, eSC) as illustrated in Fig. 5D for the phase model and in Fig. 5E for the LC model. Here, the parcellations from the same atlas also agree much better with each other than for the parcellations from different atlases. The results also demonstrate that Fit-values obtained for HO96 parcellations and the LC model [Fig. 5E] are relatively dissimilar to the other two atlases of brain parcellations. Furthermore, the similarity Fit(sFC, eSC) seems to be sensitive to the model used for simulation as illustrated in Fig. 5F. The fitting results of the LC model for S100-S600 parcellations weakly correlate with those obtained for all other parcellations for the phase model. For other atlases, the fitting results of LC model are either practically independent of those obtained for the phase model (for the Shen atlas), or even weakly anti-correlate with the other model (for HO96 atlas) even for the same brain parcellation/atlas [Fig. 5F].

Changing the brain parcellation can also influence the values of the optimal parameters, where the maximal similarity (3) is achieved. The pairwise parameter differences are illustrated in supplementary Fig. A.3 for the considered parcellations and models. Similar to the correlation between the Fit-values [Fig. 5], the parcellations from the same atlas are expected to lead to smaller variations of the optimal parameters than between those from different atlases. Interestingly, the variation of the optimal parameters is larger for the functional model fitting modality, especially, for the between-model comparison than for the structure-function correspondence. In the latter case the parameter distance between models remarkably mimics the similarity patterns of the correlation between fitting results, compare Fig. 5F and supplementary Fig. A.3F.

### 3.3 Data variables

In the next Section 3.4 we evaluate how the maximal model-data similarity (3) obtained for the optimal model parameters depends on selected statistical properties of the empirical data used for the model derivation and validation. To this end, we calculated several data variables (or indices) for each subject and fMRI scan session. For the empirical BOLD signals we calculated the standard deviation of their time fluctuations std(BOLD) averaged over all parcels aver[std(BOLD)]. Since the BOLD signals were extracted as mean signals averaged over all voxels in the parcels, the latter data variable may reflect the extent of synchronization of BOLD dynamics within the individual brain regions. Indeed, the amplitude of the mean signal is expected to increase with enhanced synchronization as the theory of synchronization implies (Kuramoto, 1984). On the other hand, calculating the variability of time fluctuations among parcels std[std(BOLD)] may give an insight into the difference of individual parcels in this respect.

**Fig. 6:**
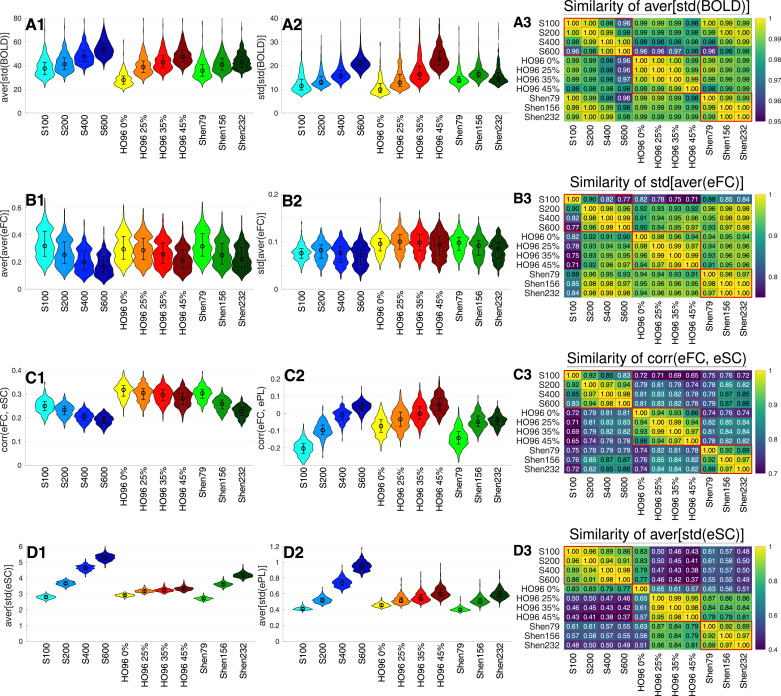
Variation of the data variables extracted for the considered brain parcellations. In columns **1** and **2**, the distributions of the data variables (indicated on the vertical axes) for all subjects/fMRI sessions are depicted versus the parcellations (indicated on the horizontal axes). In column **3**, the correspondence between the data variables among all considered parcellations is illustrated. For any two parcellations (indicated on the axes), the Pearson cross-correlation between the corresponding data variables was calculated across all subjects for (**A3**) aver[std(BOLD)], (**B3**) std[aver(eFC)], (**C3**) corr(eFC,eSC), and (**D3**) aver[std(eSC)] as indicated in the titles of the plots. The results are depicted by color, and their magnitudes are also printed in the plots. The crossed out cells indicate that the corresponding correlation does not reach the statistical significance with p<.05. The heavy red lines delineate the parcellations from the same atlas (parcellation family). (For interpretation of the references to colour in this figure legend, the reader is referred to the web version of this article.)

Smaller brain regions, e.g., for finer granularity (Schaefer, Shen) or larger probability threshold (HO96) can be suspected to be more homogeneous with respect to the BOLD dynamics. We observed that mean BOLD signals exhibit enhanced fluctuations for smaller parcels demonstrating larger standard deviation std(BOLD) [Fig. 6A1], where the distributions of aver[std(BOLD)] exhibit the behavior inverse to that of the parcels’ size versus the considered brain parcellations [Fig. 1A]. The same holds for std[std(BOLD)] [Fig. 6A2, but see Shen232]. Our calculations thus indicate that the intra-region dynamical homogeneity (synchronization) may increase together with the inter-region variability of it. However, a systematic investigation of the collective dynamics of BOLD signals within parcels is necessary to assess the intra-region dynamical homogeneity (Schaefer et al., 2018). Interestingly, the distributions of both mentioned data variables across individual subjects exhibit very similar patterns for any of the considered atlases and strongly correlate across subjects for any pair of parcellations, see Fig. 6 A3 for aver[std(BOLD)], where the minimal correlation r≈0.96 is attained for S600.

Additional data variables can be calculated from eFC by evaluation of its column-wise mean aver(eFC) and the standard deviation std(eFC), where the former represents the average functional connectivity (synchronization) of a region to the rest of the brain (i.e., other regions), and the latter stands for the extent of variation of the individual connections of a given brain region. Evaluating the mean and the standard deviation once more across all brain regions we obtain four data variables: aver[aver(eFC)], std[aver(eFC)], aver[std(eFC)], and std[std(eFC)]. The distributions of the first two are illustrated in Fig. 6B1 and B2, where the total average inter-region synchronization aver[aver(eFC)] in the brain decays with decreasing region size, which is also in agreement with the behavior observed for BOLD signals [Fig. 6A2]. The inter-region variation of the regional synchronization to the rest of the brain std[aver(eFC)] does not demonstrate very pronounced dynamics with respect to the considered parcellations [Fig. 6B2]. However, the inter-parcellation patterns of its distribution appears to be similar to those observed for the functional similarity Fit(sFC, eFC) [Fig. 3A and E]. An example of the cross-parcellation correlation for the later data variable is illustrated in Fig. 6B3, where the level of correlation is still very high with r≳0.91 except for S100 which distinguishes from the other parcellations.

Further data variables can be the extent of correlation between the empirical connectivity matrices eFC, eSC and ePL, which may influence the quality of the model fitting and are denoted as corr(eFC,eSC), corr(eFC,ePL) and corr(eSC,ePL). Examples of the distributions of these variables are shown in Fig. 6C1 and C2, where both illustrated variables apparently demonstrate a monotonic behavior with respect to the parcel size, but in opposite directions, i.e., corr(eFC,eSC) decreases, and corr(eFC,ePL) increases when the region size decays. The impact of the state-of-the-art brain parcellations on the structure-function relationship corr(eFC,eSC) was investigated by Messe (2019), and a similar global decrease in correlation with decreasing the parcellation granularity and regions size was reported. For these data variables the difference between the atlases becomes more pronounced, where the correspondence (correlation) between the data indices for the parcellations of the same atlas are stronger than for those from different atlases [Fig. 6C3] as was shown for the results of the model validation and optimal parameters [Fig. 5 and supplementary Fig. A.3].

This effect is further enhanced for the data variables derived from SC matrices, for example, for aver[std(eSC)] [Fig. 6D3]. The data variables aver[std(eSC)] and aver[std(ePL)] calculated from the eSC and ePL matrices normalized by their mean as used in the models always attain larger values for finer granularity/smaller brain regions [Fig. 6D1 and D2]. This is similar to the variables corr(eFC,ePL) [Fig. 6C2] and those derived from BOLD signals [Fig. 6A1 and A2]. This is however in contrast to the data variables calculated from eFC, where the behavior is different [Fig. 6B1, B2 and C1]. The observed increase of the average inter-region variability of SC matrices [Fig. 6D1 and D2] might be suspected when the brain is parcellated into smaller regions that stronger deviate from each other with respect to individual connectivity properties. However, a detailed investigation is necessary to clarify the underlying mechanisms of the illustrated behavior of the considered data variables [Fig. 6].

Further considered data variables in the form std[aver(·)] and std[std(·)] were calculated from the eSC and ePL matrices. The natural frequencies $f_{i}$ of the models (1) and (2) extracted from the frequency spectra of the empirical BOLD signals (see Methods) were also taken into account, and the mean $aver(f_{i})$ and the standard deviation $std(f_{i})$ were involved in the analysis.

### 3.4 Correlation between data variables and model fitting

**Fig. 7:**
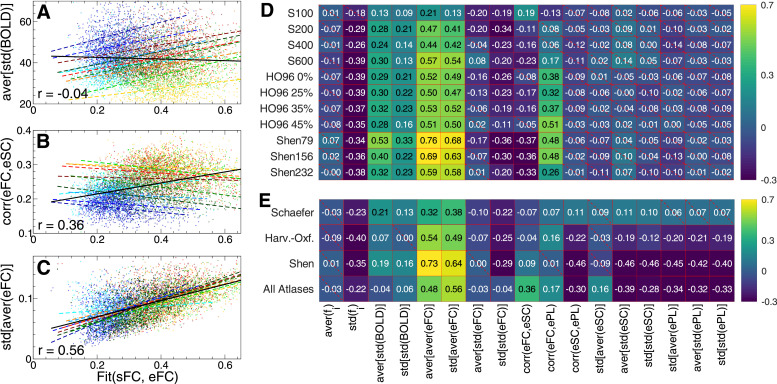
Relationship between the fitting results (3) of the phase model (1) and empirical data. (**A-C**) Scatter plots and the corresponding linear regression (straight lines) are shown for a few selected data variables from Fig. 6 indicated on the vertical axes versus the maximal similarity Fit(sFC, eFC) (horizontal axes). Each dot represents one subject/MRI session, and color corresponds to that used to differentiate between the parcellations in Fig. 6. The black solid lines depict the joint linear regressions for all data in the plots, and the joint correlations r are also indicated. (**D, E**) Pearson correlation across individual subjects between the maximal similarity Fit(sFC, eFC) and several data variables indicated on the horizontal axis. The correlation was calculated for (**D**) different individual parcellations indicated on the vertical axis and (**E**) joint data merged over a few combinations of the considered parcellations as indicated on the vertical axis: all parcellations of the Schaefer atlas, Harvard-Oxford atlas, Shen atlas and all 11 considered parcellations (last row). The correlation is depicted by color, and its magnitude is indicated in the plot. The crossed out cells indicate that the corresponding correlation does not reach the statistical significance with p<.05.

The variation of the empirical data illustrated in Fig. 6 may influence the observed variability of the modeling results [Figs. 3 and 4]. Therefore, to inquire into where the variance of the fitting results across subjects and parcellations may come from, we investigate how the discussed data variables and the maximal similarity (3) correlate with each other. Several such correlative relationships are illustrated in the scatter plots in Fig. 7A–C, where, together with linear regressions for individual parcellations (color dots and dashed lines), the joint linear regression for all data points in the plots (for all 11 parcellations) is also shown by solid black lines. The observed distinct constellations between the individual (color dashed) and joint (black solid) regression lines can be used to differentiate between a few classes of the data variables with respect to their relationships to the overall model fitting. For example, for the data variable aver[std(BOLD)] [Fig. 7A] we found that the joint correlation indicated in the plot appears to be much smaller than the correlative relationships obtained separately for each individual parcellation. Therefore, the variation of the mentioned data variable can relatively well account for the variability of the model fitting across individual subjects for a given parcellation, i.e., for the intra-parcellation inter-subject variance. However, its explanatory power for the variation of Fit(sFC, eFC) across considered parcellations is limited. We may thus refer to such data indices as intra-parcellation variables.

Another class of the data variables can be illustrated by the data index corr(eFC,eSC) [Fig. 7B]. Here, the joint correlation between the empirical data and the model goodness-of-fit across subject data from different parcellations can be much higher than the correspondence across subjects within individual parcellations. In the considered example, the across-subject correlations between the empirical data and results of the model fitting are mostly small and negative for individual parcellations. Therefore this data variable can hardly explain the variance of the model fitting across subjects for a given brain parcellation. Nevertheless, the joint correlation for the data merged over all parcellations is much stronger contributing to our understanding of the variance of the fitting results across different parcellations. We may thus refer to such data indices as inter-parcellation variables.

For some other data variables, for example, for std[aver(eFC)] the joint correlation is comparable to the relatively large correlations for individual parcellations [Fig. 7C]. The explanatory power of such variables can thus be extended from single to many parcellations. This indicates that such data variables can therefore well account for both the variability of the model fitting across subjects within individual parcellations and the differences of Fit-values across parcellations. We may thus refer to such data indices as the variables of both intra- and inter-parcellation types.

The correlations across subjects and scanning sessions between the similarity Fit(sFC, eFC) and all mentioned data variables are shown in Fig. 7D for all considered parcellations. One in particular observes that there are several data variables that only weakly correlate with Fit(sFC, eFC), which may indicate that the results of the model fitting may little depend on them. Such conclusion could be made for the mean of the natural frequencies $aver(f_{i})$, average variability of eFC aver[std(eFC)] (except for S100 and S200), and also for the data indices derived from eSC and ePL. Notably, the extent of the empirical structure-function relationship corr(eFC,eSC) also little correlates with the correspondence between simulated and empirical functional data, see also Fig. 7B. Put otherwise, increasing/decreasing the agreement between the empirical structure (eSC) and function (eFC) seems not to essentially influence the quality of the model fitting (the similarity between sFC and eFC) or may even have a negative effect. This takes place in spite of that the network model is constructed from eSC and its output is compared with eFC.

Other data variables consistently exhibit (anti-)correlation with Fit(sFC, eFC) ranging from moderate to relatively strong for most of the parcellations. This for instance applies to the spread of the natural frequencies $std(f_{i})$, amplitude aver[std(BOLD)] of the BOLD signals and some properties of eFC [Fig. 7D]. These data variables may be used to provide an initial guess of the pattern of the functional model fitting for new subjects that supposed to be included in the analysis. However, the correlation between eFC and ePL matrices corr(eFC,ePL) seems to have a different impact on the model validation for different atlases, where Fit(sFC, eFC) is practically independent of this data index for the Schaefer atlas, which is distinct for other atlases [Fig. 7D]. Such effects may also be useful for understanding the observed differences in the quality of the model fitting for individual subjects and may also be applied for explaining the impact of the considered brain parcellations on the model fitting [Fig. 3A].

**Fig. 8:**
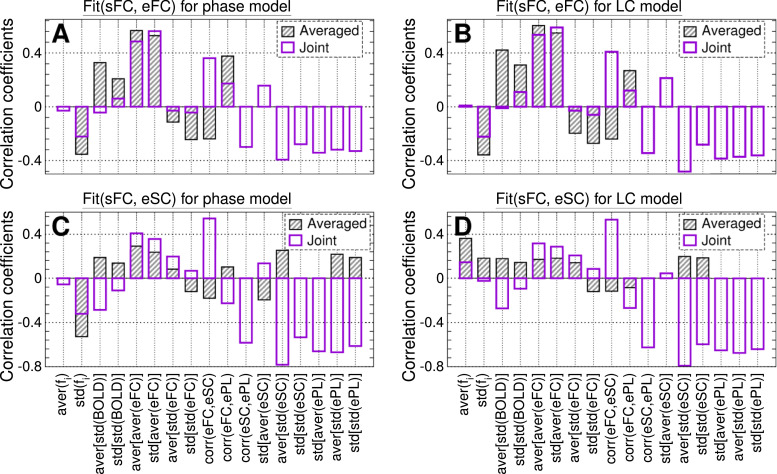
Correlation between the Fit-values (3) and data variables from Fig. 7 jointly for all considered brain parcellations. For the data variables indicated on the horizontal axes, the joint correlation for the data merged over all considered parcellations [Fig. 7E, last row] is depicted by empty bars. The hatched bars represent the correlation for individual parcellations from Fig. 7D averaged over all parcellations and significant values (i.e., excluding the crossed out cells in Fig. 7D) as indicated in the legends. The data is shown for (**A, B**) functional fitting Fit(sFC, eFC) and (**C, D**) structure-functional fitting Fit(sFC, eSC), and for (**A, C**) phase model (1) and (**B, D**) LC model (2) as indicated in the titles of the plots.

The above classification of the data variables with respect to their intra- or inter-parcellation correlative relationships with the modeling results [Fig. 7A–C] can be evaluated by comparing the individual correlations in Fig. 7D to the joint correlation calculated for the data merged over the considered parcellations for simultaneous analysis. This is illustrated in Fig. 7E for the phase model and functional model fitting. More systematic comparison of the individual and joint correlations between the results of the model fitting (3) and the data variables is summarized in Fig. 8 for both models (1) and (2) and both fitting modalities Fit(sFC, eFC) and Fit(sFC, eSC). Much larger individual (joint) correlation than the joint (individual) one is indicative for an intra- (inter-) parcellation data variable.

The constellation obtained for the phase model [Fig. 8A] is well preserved also for the LC model [Fig. 8B, see also supplementary Fig. A.4 for individual and joint correlations]. The correlation patterns are different for the structure-functional fitting modality [Fig. 8C and D], where the results obtained for the phase and LC models may deviate from each other, see also supplementary Fig. A.5 for individual and joint correlations for the structure-functional fitting modality Fit(sFC, eSC). Although most of the considered data indices exhibiting large correlation are of inter-parcellation type [Fig. 8], still there are a few data variables of intra-parcellation type like $std(f_{i})$, aver[std(BOLD)] or std[std(eFC)] for the functional similarity Fit(sFC, eFC) or $std(f_{i})$ (phase model) and $aver(f_{i})$ (LC model) for Fit(sFC, eSC). The most pronounced data variables of both types for Fit(sFC, eFC) are given by the total average inter-region synchronization aver[aver(eFC)] or inter-region variation of the regional synchronization std[aver(eFC)] [Fig. 8A,B].

### 3.5 Multiple linear regression model

**Fig. 9:**
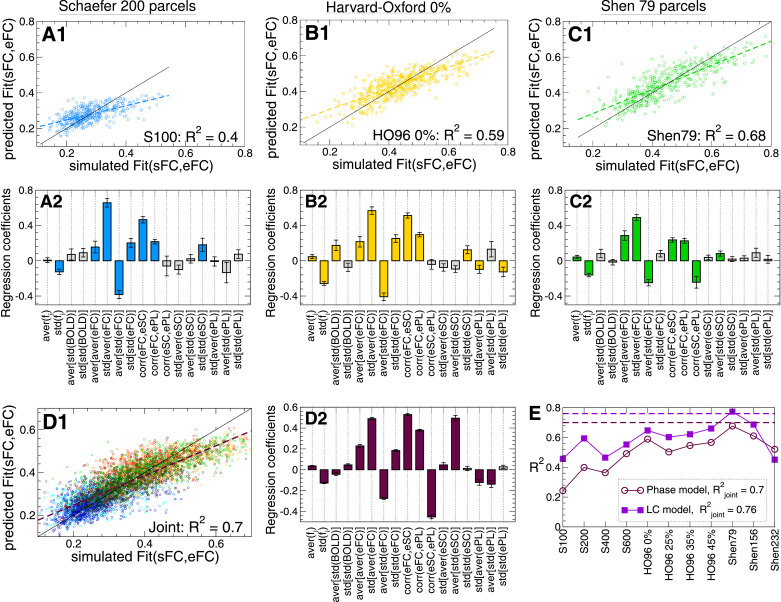
Modeling the maximal similarity Fit(sFC, eFC) by the multiple linear regression (MLR) model with data variables from Fig. 7 as independent variables. (**A1** - **D1**) Scatter plots with regression lines of the Fit-values predicted by MLR versus Fit(sFC, eFC) obtained by simulations of the phase model (1). The diagonals are depicted by thin black lines for comparison. (**A2** - **D2**) The corresponding regression coefficients with the standard deviation for z-scored data obtained from the model fitting for parcellations (**A**) S200 and (**B**) HO96 0%, (**C**) Shen79 and (**D**) for joint data merged over all considered parcellations as indicated in the corresponding scatter plots. The gray bars indicate the regression coefficients, where the statistical significance with p<.05 was not achieved. The fractions of the explained variance $R^{2}$ are also shown in the scatter plots and in plot (**E**) for all individual parcellations for both phase and LC models as indicated in the legend. The dashed lines depict $R^{2}$ for the joint data also indicated in the legend.

The variation of the model fitting across subjects and brain parcellations can be investigated by combining several data variables in a MLR model, where they serve as independent (explanatory) variables, and the maximal similarity Fit(sFC, eFC) is the MLR output, i.e., the dependent variable. The calculated data variables can be used in the MLR model to evaluate which variation of the Fit-values across subjects and parcellations can be explained by the individual empirical data used for the model derivation and validation. The results of such a regression with respect to all data variables [Fig. 7] are illustrated in Fig. 9 for investigated individual parcellations as well as for the joint data merged over all parcellations. The fraction of the explained variance increases when more data variables get involved in the regression, see Fig. 9A–C and compare the indicated $R^{2}$-values to the correlation coefficients in Figs. 7 and 8. The results of the model fitting for the anatomical Harvard-Oxford and the functional Shen atlases seem to be somewhat better explained by the empirical data used for the model derivation than for the functional Schaefer atlas [Fig. 9E, but see Shen232 for LC model]. The strongest regression results are obtained for the joint regression for the data merged over all considered parcellations [Fig. 9D and E].

**Fig. 10:**
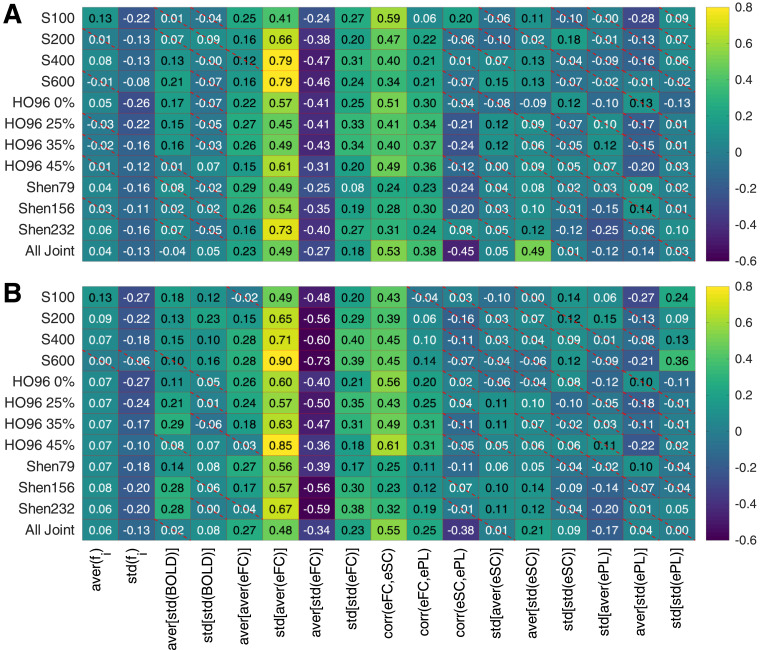
Regression coefficients of the MLR model for Fit(sFC, eFC), for all considered parcellations including the joint data as indicated on the vertical axes and for (**A**) phase model and (**B**) LC model. The values are depicted by color, and they magnitudes are shown in the plots. The crossed out cells indicate that the corresponding coefficient does not reach the statistical significance with p<.05.

The weights of the discussed data variables within the maximal similarity Fit(sFC, eFC) as reflected by the regression coefficients [Fig. 9 A2-D2] highlight several data variables that are of importance for understanding of the modeling results. All regression coefficients for the interdependency between Fit(sFC, eFC) and the data variables are shown in Fig. 10 for both models including the case of joint data (last rows in the plots). Comparing the obtained results for individual parcellations and models, we observe that the regression coefficients well agree between the two models. There are several data indices that consistently and strongly contribute to the Fit-values and seem to have a major impact on the model fitting for many parcellations, see Figs. 9 and 10. In particular, the variables std[aver(eFC)], aver[std(eFC)] and corr(eFC,eSC) have the most notable regression coefficients. At the level of individual parcellations, there is also a minor impact of other variables, for example, the natural frequencies $std(f_{i})$, average total connectivity aver[aver(eFC)] and its variability std[std(eFC)] as well as structure-function relationship with ePL matrix corr(eFC,ePL). For the inter-parcellation variance of Fit(sFC, eFC), additional variables can be taken into account, that are corr(eFC,ePL) and aver[std(eSC)] as suggested by the MLR model [Fig. 10].

Similar results can also be obtained for the structure-functional model fitting and the maximal similarity Fit(sFC, eSC) [supplementary Fig. A.6]. Here we however find that Fit(sFC, eSC) less consistently depends on the data variables over individual parcellations and with a reduced agreement between different models as reflected by the MLR coefficients. The only data indices that reliably contribute to the inter-individual variation of the Fit-values for most of the parcellations are those extracted from the natural frequencies $aver(f_{i})$ and $std(f_{i})$, while the latter is again less reliable for the LC model [supplementary Fig. A.6 A and B]. The fractions of the Fit(sFC, eSC) variance explained by the data variables for individual parcellations is reduced as compared to the functional model fitting [compare Fig. 9E and supplementary Fig. A.6 D]. However, the inter-parcellation variance as reflected by the joint data can still be relatively well accounted for by the empirical data [supplementary Fig. A.6 C], and the largest MLR coefficients of the joint data for both models are obtained for the structural connectome eSC and ePL [supplementary Fig. A.6 A and B].

### 3.6 Group-level inter-parcellation variations

In the previous sections the interdependence between the results of the model validation and empirical data were evaluated by correlation of the Fit-values with the data variables across individual subjects either for any parcellation separately or for joint data merged over all considered parcellations. While the former approach investigates the inter-subject intra-parcellation variance, the latter also considers the variation of the variables among parcellations. The inter-parcellation variation of the fitting results can also be addressed at the group level only, i.e, separated from the inter-subject variations. This can be accomplished when the data calculated for individual subjects is compressed into single values, for example, to medians, see Figs. 3 and 4. The behavior of the group-averaged values across individual parcellations can provide an informed expectation on how a given parcellation may in average influence the considered variables, for example, the Fit-values or the data indices.

**Fig. 11:**
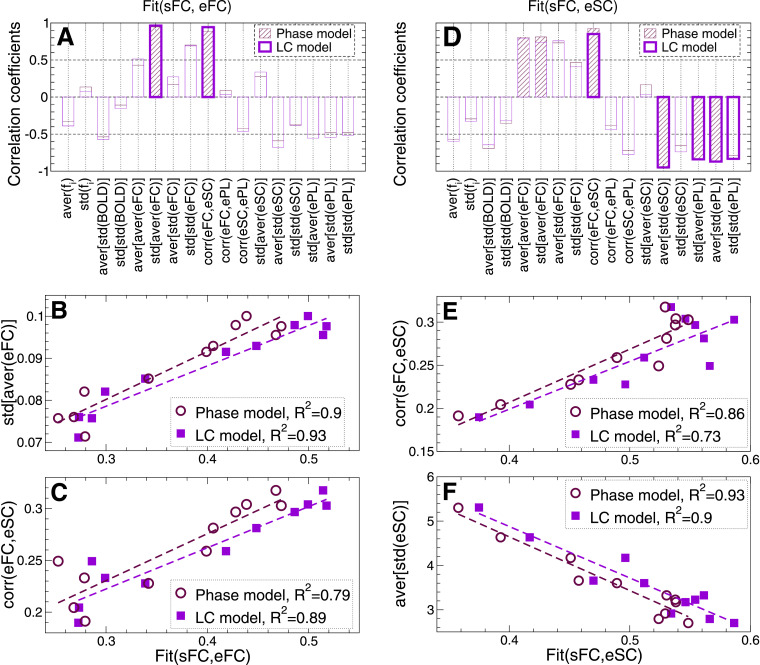
Correlation between the results of the model fitting and data variables at the group level. (**A, D**) Pearson correlation across pacellations between the medians evaluated over all subjects of the data variables and the corresponding medians of (**A**) Fit(sFC, eFC) and (**D**) Fit(sFC, eSC). The hatched bars for the phase model and empty violet bars with heavy borders for the LC model stand for statistically significant (p<.05) correlation coefficients. (**B,C,E,F**) Scatter plots of the medians of the data variables versus (**B, C**) Fit(sFC, eFC) and (**E, F**) Fit(sFC, eSC) with the corresponding regression lines. Each plot symbol corresponds to one of the considered parcellations. The fractions of the explained variance (squared correlation) for both models are indicated in the legends.

In this section we correlate the medians of the Fit-values and the considered data variables across parcellations. The results of the calculations are illustrated in Fig. 11. Several data variables exhibit strong correspondence with the Fit-values for both models. However, only a few of them are significantly correlated as indicated by hatched bars for the phase model and empty bars with heavy borders for the LC model [Fig. 11A and D]. For the functional modal fitting, only two data indices std[aver(eFC)] and corr(eFC,eSC) significantly and strongly contribute to the inter-parcellation variance of Fit(sFC, eFC) at the group level for both models [Fig. 11A], see also Fig. 11B and C for the corresponding scatter plots, where the fraction of the explained variance can reach 93%.

For the structure-functional model fitting, more data variables significantly correlate with the maximal similarity Fit(sFC, eSC) [Fig. 11D]. However, only four of them fulfill this requirement for both models simultaneously: corr(eFC,eSC) that also contributes to Fit(sFC, eFC), as well as data variables aver[std(eSC)], std[aver(ePL)] and aver[std(ePL)] calculated from the structural connectome as given by eSC and ePL matrices. Again, the fraction of the explained variance can reach 93% for the data index calculated from eSC, see Fig. 11D-F also for the corresponding scatter plots. Interestingly, for the structure-functional fitting modality also the data indices derived from eFC matrices seem to significantly contribute to the fitting values Fit(sFC, eSC) for the phase model [Fig. 11D], although the corresponding p-values are close to the significance threshold of 0.05 after correction for multiple comparisons.

## 4 Discussion

We investigated the impact of data parameters used for the pre-processing of the empirical neuroimaging data on the structure and dynamics of whole-brain dynamical models derived from and validated against empirical data. In this study we focused on brain parcellations and considered three brain atlases as defined by the functional Schaefer atlas with 100, 200, 400 and 600 cortical regions (Schaefer et al., 2018), functional Shen atlas with 79, 156 and 232 cortical regions (Shen et al., 2013), and the anatomical Harvard-Oxford atlas of 96 cortical regions with a few thresholds of the maximal probability (Desikan et al., 2006) that also influenced the region size. Here we did not aim to suggest an optimal atlas, which is a complex task given the numerous existing parcellation approaches, brain atlases and multiplicity of possible optimization criteria. Instead, we illustrated possible effects that the considered brain parcellations can have on the modeling results. For this we analyzed the results of the model validation for two fitting modalities as given by the maximal similarities Fit(sFC, eFC) and Fit(sFC, eSC) and for two models of coupled phase and limit-cycle oscillators. We also suggested an approach to account for the parcellation-induced inter-subject and inter-parcellation variability of the fitting results.

We compared the distributions of the Fit-values and the corresponding optimal parameters for individual subjects and reported on pronounced differences in the model fitting between the considered brain parcellations. In particular, Fit(sFC, eFC) for the Schaefer atlas is much smaller than that for the Harvard-Oxford and Shen atlases [Fig. 3]. The latter atlases seem to provide appropriate parcellations for high correspondence between simulated and empirical functional data. The better fitting for HO96 0% as compared to S100 was also observed for the model of coupled phase oscillators simulating the high-frequency electrical activity of brain regions in α and γ frequency bands (Manos et al., 2019). For the structure-functional model fitting Fit(sFC, eSC) the situation is different, and the difference between the atlases is less pronounced [Fig. 4].

We demonstrated that the best correspondence Fit(sFC,eFC) between simulated and empirical FCs was achieved at 200 parcels for the Schaefer atlas [Fig. 3] suggesting that an optimal spatial scale may exist, see also (Arslan, Ktena, Makropoulos, Robinson, Rueckert, Parisot, 2018, Proix, Spiegler, Schirner, Rothmeier, Ritter, Jirsa, 2016). However, the best functional model fitting for the other brain atlases was achieved at the coarsest granularity (Shen atlas) or smallest probability threshold (Harvard-Oxford atlas), where the parcel size is maximal. On the other hand, the maximal values of the structure-functional model fitting Fit(sFC, eSC) were achieved at the largest region size for the Schaefer and Shen atlases [Fig. 4]. For the Harvard-Oxford atlas, Fit(sFC, eSC) exhibited either non-monotonic behavior with the optimal probability thresholds at 25%–35% for the phase model or even monotonically increased for the LC model when the region size decreased. We thus observed a remarkable exchange of the distribution patterns of Fit(sFC, eFC) and Fit(sFC, eSC) between the Schaefer and Harvard-Oxford atlases and different behavior of the Fit-values with respect to the parcel size. These findings complicate the problem of the optimal spatial scale of brain parcellation.

The corresponding distributions of the optimal model parameters however manifest very similar shapes for the same fitting modality also for different atlases and parcellations, but differ across fitting modalities [Figs. 3 and 4]. In particular, the optimal parameters for Fit(sFC, eFC) are strongly concentrated towards zero delay, whereas the structure-function correspondence Fit(sFC, eSC) for many subjects was also achieved for large delay, especially, for the phase model. This is accompanied by a narrow interval of the coupling strength in the latter case, whereas this parameter can broadly be distributed for the functional fitting, especially, for the Shen atlas and phase model. Therefore, the direct modeling of the resting-state BOLD dynamics by slowly oscillating phase or limit-cycle oscillators can safely be performed by systems without delay (Deco, Cruzat, Cabral, Tagliazucchi, Laufs, Logothetis, Kringelbach, 2019, Deco, Kringelbach, Jirsa, Ritter, 2017, Ponce-Alvarez, Deco, Hagmann, Romani, Mantini, Corbetta, 2015), however, only for the fitting of the simulated and empirical functional data.

The impact of the brain parcellations on the model validation can be investigated by evaluation of how the fitting results Fit(·,·) calculated for individual subjects and a given parcellation agree with those found for other parcellations. We thus correlated Fit-values for different parcellations across subjects and calculated the distance between the corresponding optimal model parameters. It appeared that Fit-values for the parcellations within the same atlas better correlate with each other than across different atlases for both fitting modalities Fit(sFC, eFC) and Fit(sFC, eSC) and both considered models [Fig. 5]. The same is true for the distance between the optimal parameters, where they less deviate from each other for the parcellations from the same atlas than between atlases [supplementary Fig. A.3]. It is interesting to note that neither different numbers of brain regions for the Schaefer and Shen atlases nor different level of thresholding for the Harvard-Oxford atlas can cause differences in the cross-subject correspondence in the model fitting larger than those between different atlases even for parcellations with similar region size. The inter-atlas differences cannot simply be reduced to differentiation between anatomical and functional parcellation approaches considered in this study. This indicates that a parcellation family (atlas) shares some particular properties that are reflected in the results of the model fitting and preserved even for varying other “internal” parcellation parameters (e.g., granularity or probability threshold affecting region size). This conclusion is also preserved for between-model comparison for Fit(sFC, eFC), whereas the structure-functional fitting results Fit(sFC, eSC) obtained for the LC model demonstrated enhanced sensitivity, especially, for the Harvard-Oxford atlas [Fig. 5].

To understand the origin of the observed variation of the fitting results across subjects and brain parcellations, we suggested to evaluate how the Fit-values depend on a few data variables (or data indices) reflecting some statistical properties of the empirical data used for the model derivation and validation. The performed regressive analysis between Fit-values and data variables suggested that the latter can be split into a few classes depending on their explanatory power for (*i*) inter-subject Fit-variance for individual parcellations; (*ii*) inter-subject Fit-variance across parcellations for joint data; and (*iii*) both inter-subject Fit-variance within individual parcellations and across them [Figs. 7 and 8].

The bivariate analysis provided correlation between Fit-values and individual data variables, where the squared correlation with Fit(sFC, eFC) across subjects can reach $R^{2}=64\%$ for individual parcellations and 35% for joint data merged over all considered parcellations [Fig. 7 and supplementary Fig. A.4]. For the structure-functional model fitting Fit(sFC, eSC), this quantity may range up to 40% for the variance across subjects for individual parcellations and about 62% for joint data [supplementary Fig. A.5]. The inter-subject fluctuations of the Fit-values may be better accounted for if several data variables are used in the MLR model [Fig. 9]. With the multivariate approach, the inter-subject variance of Fit(sFC, eFC) and Fit(sFC, eSC) can be explained up to 77% and 56% within individual parcellations and up to 76% and 77% for joint data, respectively [Fig. 9 and supplementary Fig. A.6]. Finally, if the variance of the fitting results across parcellations is considered at the group level only (as medians), the individual data variables correlate with the fitting values up to $R^{2}=93\%$ [Fig. 11].

Evaluating the effect that a given parcellation can have on the data variables, which reliably, strongly and significantly correlate with the fitting values as investigated in this study, can help to explain and predict the results of the model fitting before involving computationally expensive model simulations. This can be addressed by investigating the properties of the empirical data extracted for varying brain parcellation. Decrease of the region size due to finer granularity or larger cutting threshold seems to cause two main effects, where both (*i*) the intra-region dynamical homogeneity and (*ii*) inter-region heterogeneity appeared to increase. This can be concluded from the behavior of the mean BOLD signals of the brain regions and the extent of total synchronization between regions aver[aver(eFC)] [Fig. 6]. The inter-region heterogeneity seems to increase for smaller regions also for the structural connectome as demonstrated by the data variables derived from eSC, ePL. Here, the empirical structure-function relationship corr(eFC,eSC) decays with decreasing region size as was also reported by Messe (2019). It is interesting to note that the correspondence between structure and function is larger for the Harvard-Oxford atlas and the coarsest granularity of the Shen atlas as compared to the Schaefer atlas. Investigation of the impact of brain parcellations on the data variables should also take into account inter-subject spatial variability (shape and location) of brain regions, which seems to influence the cross-subject variability of the resting-state fMRI data and functional connectivity (Bijsterbosch, Woolrich, Glasser, Robinson, Beckmann, Van Essen, Harrison, Smith, 2018, Kong, Li, Orban, Sabuncu, Liu, Schaefer, Sun, Zuo, Holmes, Eickhoff, Yeo, 2018).

Among the considered data variables only a few indeed exhibit relatively strong interdependencies with the Fit-values across subjects and parcellations [Fig. 7, Fig. 8, Fig. 9, Fig. 10, Fig. 11]. These sets of the data variables may vary for different fitting modalities and models. Here, the behavior of corr(eFC,eSC) is of special interest, because the empirical structure-function correspondence might be suspected to underlie the model fitting results Fit(sFC, eFC) and Fit(sFC, eSC). Our investigations however showed that corr(eFC,eSC) only weakly anti-correlate with Fit-values across subjects for practically all of the considered parcellations [Fig. 7 and supplementary Figs. A.4 and A.5]. On the other hand, corr(eFC,eSC) relatively strongly correlates with the Fit-values for joint data [Fig. 8] and can thus potentially be used to explain the variation of the fitting results between atlases, especially, if the prediction is performed at the group-averaged level [Fig. 11]. In addition to the variable of the structure-function relationship, the attention might also be paid to other data indices including the average BOLD amplitude aver[std(BOLD)], the total synchronization aver[aver(eFC)], variability of the regional synchronization std[aver(eFC)] and the average variability of inter-region structural connectivity aver[std(eSC)]. Further data indices derived from the path length matrices ePL and natural frequencies $f_{i}$ might also be of importance, especially, for the structure-functional model fitting Fit(sFC, eSC).

Examining the similarities and differences in the interdependencies between the Fit-values and data variables for individual parcellations, joint and group-averaged data we may reveal the properties that are crucial for understanding the impact of brain parcellations on the empirical and simulated data. In this study we presented several interesting observations that require further detailed investigation and explanation, which could contribute to the mechanisms influencing the modeling results. In the first turn, this concerns the counter-intuitive negative dependencies (or their absence) between the empirical structure-function relationship and fitting results at the subject level in contrast to the group level as discussed above. Understanding the relationship between the fitting results and other data variables, especially, for different fitting modalities is also important. In this respect, we observed that the parcellation-induced variability of the structure-functional model fitting across subjects appears to be sensitive to the model and parcellation considered, whereas the functional fitting is relatively robust against different parcellations and models [Fig. 5]. Another issue relates with the mechanism of how the parcellation granularity and region size influence the correspondence between empirical and simulated functional and structural connectomes, which was found to be a difficult problem already for empirical data (Messe, 2019). We suggested to address these questions by separating the inter-subject and inter-parcellation variability of the modeling results and their investigation by inspecting the data indices computed from the empirical data. This approach needs to be confirmed and refined for more parcellations, models and datasets.

In this study we used the HCP dataset, where the data quality is close to a perfect physiological noise reduction. Examining different processing strategies and their parameters can be an object of investigation for further studies. In addition, other measures of similarity between simulated and empirical data can be used to verify the obtained results, for example, the amount of metastability or similarity between simulated and empirical dynamic FC (Deco et al., 2017). The generalization of the reported results should be based on profound hypothesis testing involving sophisticated statistical methods for evaluation and comparison of correlation (Wilcox and Rousselet, 2018). On the other hand, instead of similarity measures based on correlative relationships one may utilize linear models that could resolve some issues connected with heteroscedasticity of the data (Thirion et al., 2015). Some other data indices may be calculated from empirical data. For example, the graph-theoretical network properties of the empirical connectome may be involved in the analysis as well (Rubinov and Sporns, 2010). Selecting and investigating a few most important data variables with respect to their impact on the modeling results, and on a data-driven analysis of brain networks, could advance our understanding of the results’ variability across subjects and parcellations.

## Data and code availability statement

MRI data used in this study are publicly available from ConnectomeDB (db.humanconnectome.org). The code used for the simulation of the brain network dynamics can be found here: https://gitlab-public.fz-juelich.de/brainmodelling/resting-state-atlases.

## Ethics statement

Human subjects: HCP data (https://www.humanconnectome.org/, (Van Essen et al., 2013)) were acquired using protocols approved by University institutional review board (Mapping the Human and Heritability; IRB # 201204036). Informed consent was obtained from subjects.

## CRediT authorship contribution statement

**Oleksandr V. Popovych:** Conceptualization, Resources, Data curation, Software, Formal analysis, Validation, Investigation, Visualization, Methodology, Writing - original draft, Writing - review & editing. **Kyesam Jung:** Data curation, Software, Validation, Methodology, Writing - review & editing. **Thanos Manos:** Conceptualization, Resources, Data curation, Methodology, Writing - review & editing. **Sandra Diaz-Pier:** Resources, Software, Methodology, Writing - review & editing. **Felix Hoffstaedter:** Data curation, Software, Methodology, Writing - review & editing. **Jan Schreiber:** Data curation, Software, Methodology, Writing - review & editing. **B.T. Thomas Yeo:** Resources, Validation, Methodology, Writing - review & editing. **Simon B. Eickhoff:** Conceptualization, Resources, Supervision, Funding acquisition, Validation, Methodology, Project administration, Writing - review & editing.

## Declaration of Competing Interest

The authors have declared that no competing interests exist.

## Appendix A Supplementary materials

Supplementary Data S1 Supplementary Raw Research Data. This is open data under the CC BY license http://creativecommons.org/licenses/by/4.0/ Supplementary Data S1
