# Supplementary material for: Inter-subject and inter-parcellation variability of resting-state whole-brain dynamical modeling
Source: Neuroimage. 2021 Aug 1;236:118201. doi: 10.1016/j.neuroimage.2021.118201 (PMC8271096; doi:10.1016/j.neuroimage.2021.118201)
Supplement: Supplementary Data S1 — Supplementary Raw Research Data. This is open data under the CC BY license http://creativecommons.org/licenses/by/4.0/ [file mmc1.pdf]

Appendix A. Supplementary Figures

**Parcellation**	Phase oscillators **Fit(sFC, eFC)**	Phase oscillators **Fit(sFC, eSC)**	LC oscillators **Fit(sFC, eFC)**	LC oscillators **Fit(sFC, eSC)**
S100	0.25 ± 0.07	0.52 ± 0.05	0.28 ± 0.08	0.56 ± 0.04
S200	17 ± 24 %	-14 ± 8 %	7 ± 17 %	-20 ± 5 %
S400	10± 23 %	-27 ± 7 %	-4 ± 17 %	-30 ± 5 %
S600	14± 26 %	-33 ± 6 %	-4 ± 17 %	-37 ± 5 %
HO96 0%	76 ± 47 %	3 ± 10 %	75 ± 44 %	-6 ± 11 %
HO96 25%	72 ± 49 %	5 ± 10 %	73 ± 43 %	-3 ± 11 %
HO96 35%	69 ± 46 %	5 ± 11 %	68 ± 42 %	-1 ± 12 %
HO96 45%	61 ± 42 %	3 ± 12 %	59 ± 39 %	1 ± 12 %
Shen79	80 ± 56 %	8 ± 11 %	80 ± 48 %	6 ± 9 %
Shen156	61 ± 46 %	-7 ± 9 %	52 ± 36 %	-11 ± 7 %
Shen232	43 ± 37 %	-15 ± 8 %	24 ± 29 %	-13 ± 8 %

Table A.1: Relative change of the best model fit (mean ± standard deviation, in %) with respect to the case S100 (the corresponding fitting values for S100 are included in the top row) for the two model fitting modalities Fit(sFC,eFC) [Fig. 3] and Fit(sFC, eSC) [Fig. 4], and the two considered models of coupled phase and limit-cycle (LC) oscillators as indicated in the first row.

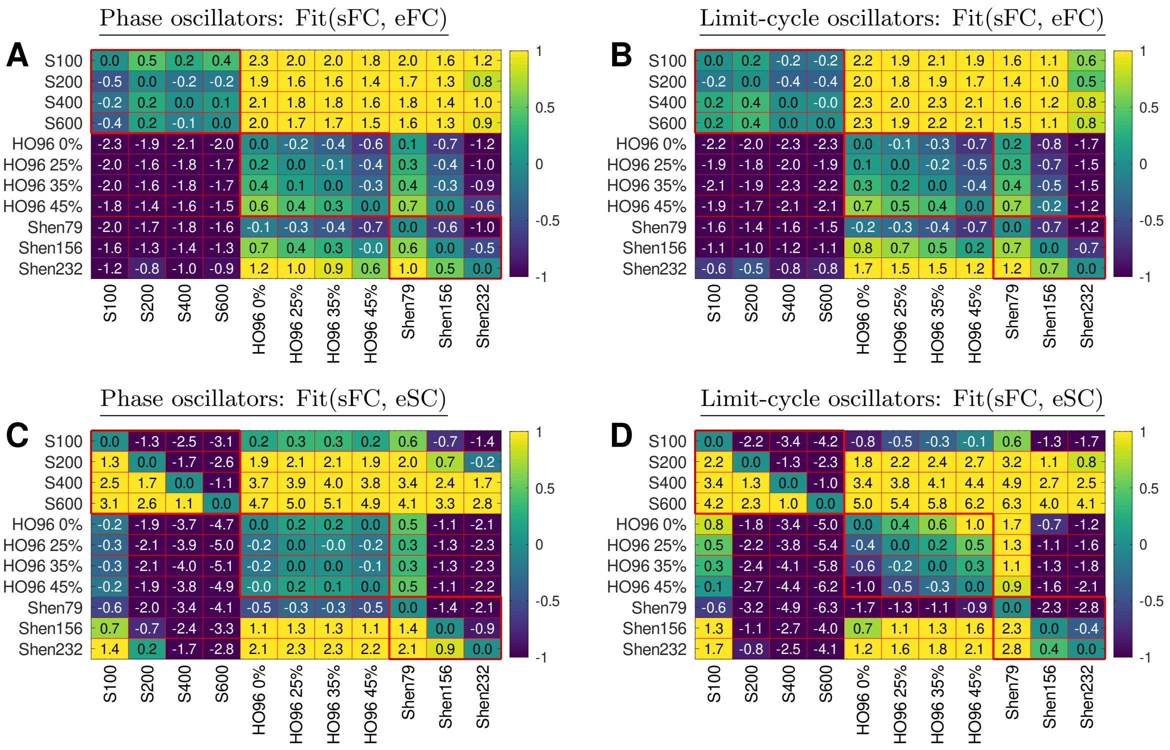

Figure A.1: Effect size as given by Cohen's d values of the paired comparison across subjects of the fitting values (**A, B**) Fit(sFC, eFC) and (**C, D**) Fit(sFC, eSC) between different parcellations and for (**A, C**) phase model and (**B, D**) limit-cycle model. The differences $\mathrm{Fit}^{(\mathrm{column})} - \mathrm{Fit}^{(\mathrm{row})}$ were examined for the Fit-values calculated for the parcellations indicated on the horizontal axes (columns) and vertical axes (rows), respectively. The corresponding cells of the table contain the calculated Cohen's d values explicitly indicated and depicted by color. The corresponding p-values of the paired Wilcoxon signed-rank test are illustrated in Figs. 3 and 4.

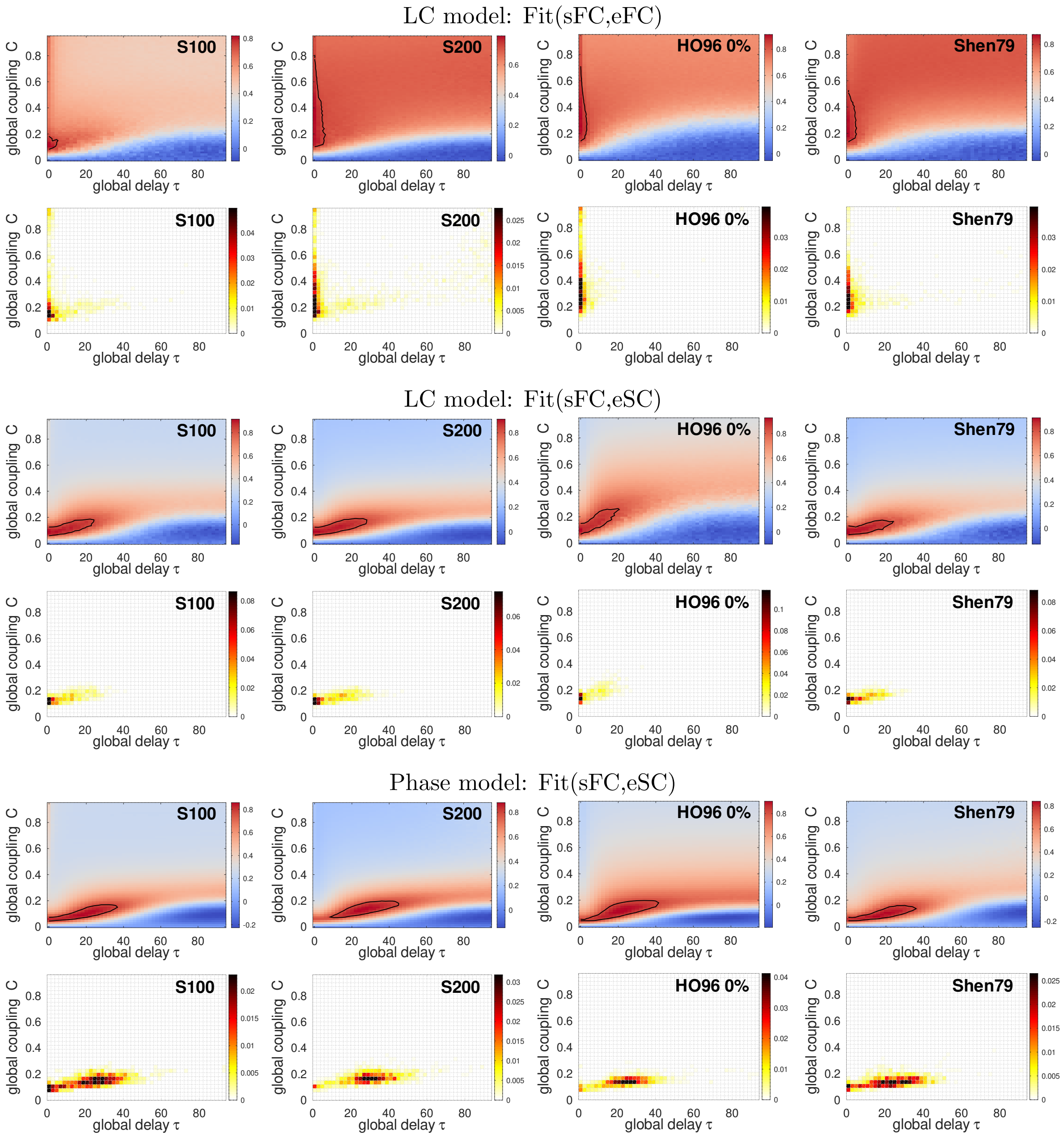

Figure A.2: Examples of parameter planes and distributions of the optimal parameters of similarity $corr(\text{sFC}, \text{eFC})$ (two upper rows) and $corr(\text{sFC}, \text{eSC})$ (four lower rows) for limit-cycle model (2) and phase model (1) as indicated on top of the plots and for parcellations indicated in the plots. The colored panels (uneven rows) are parameter planes of the model-data similarity averaged over all subjects/fMRI scans, where individual planes were first normalized by their maximal values. The black curses delineate the contour lines of 90% of maximum. The distribution of the optimal parameters (2Dim histograms in even rows) were calculated from all 5 values of the largest similarity values detected for every individual parameter plane, see Fig. 2A and D (white circles and blue diamonds).

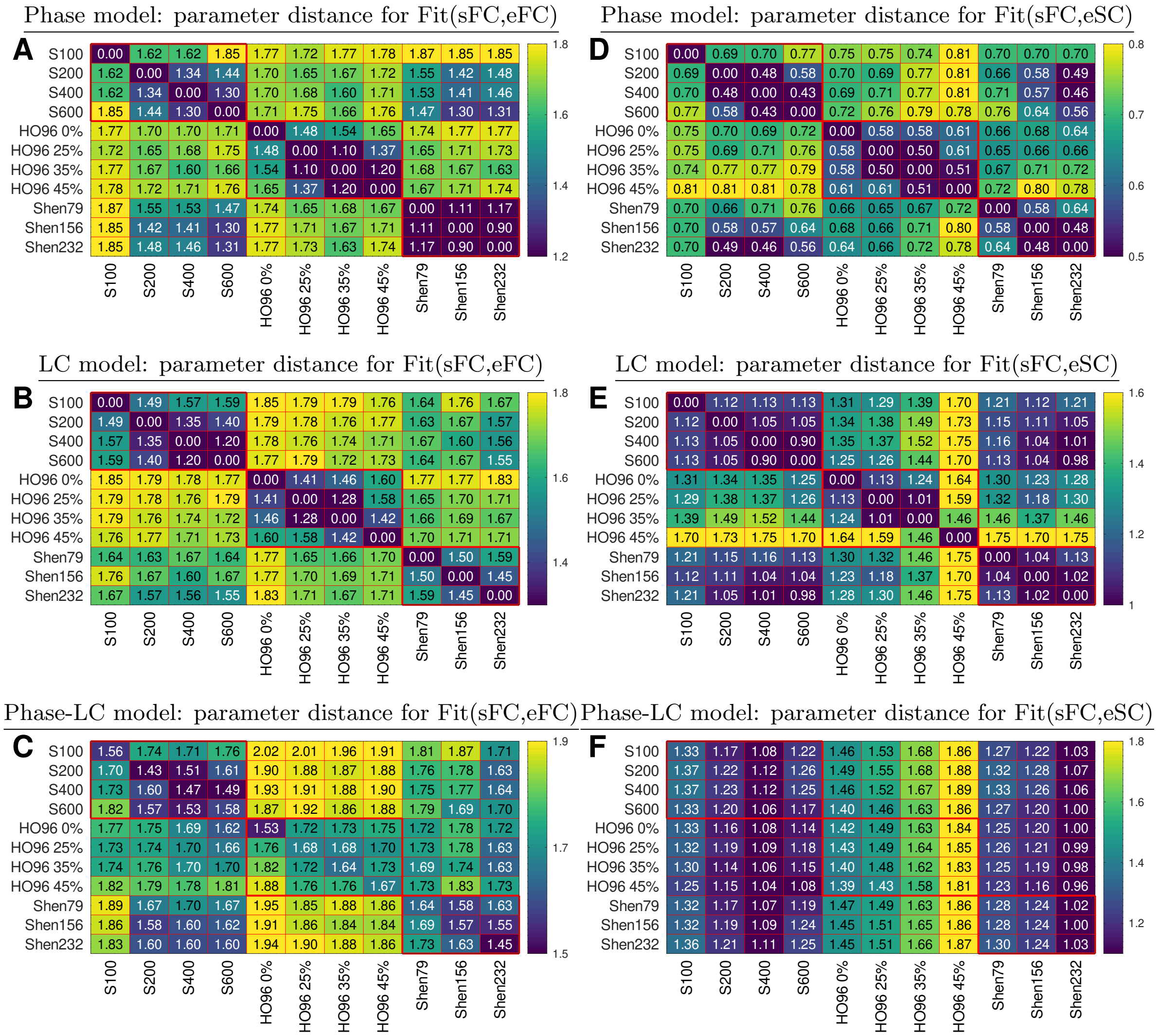

Figure A.3: Correspondence between the inter-individual variation of the optimal model parameters where the best correspondence between simulated and empirical data is achieved for the considered parcellations and models. Relative differences between the optimal parameters $P_1 = (\tau_1, C_1)$ and $P_2 = (\tau_2, C_2)$ for a given subject/fMRI session and for two different parcellations were calculated as $\left\langle \|(P_1 - P_2) / [(P_1 + P_2)/2]\| \right\rangle$ with element-wise division and averaging $\langle\cdot\rangle$ over all subjects/fMRI sessions. The calculation results performed for any two parcellations (indicated on the axes) are illustrated for (**A - C**) Fit(sFC, eFC) and (**D - F**) Fit(sFC, eSC), and for (**A, D**) phase model and (**B, E**) LC model. In plots (**C** and **F**) the correspondence between the phase model (parcellations on the vertical axes) and LC model (parcellations on the horizontal axes) is illustrated. The results are depicted by color, and their magnitudes are indicated in the plots. The heavy red lines delineate the parcellations from the same atlas (parcellation family).

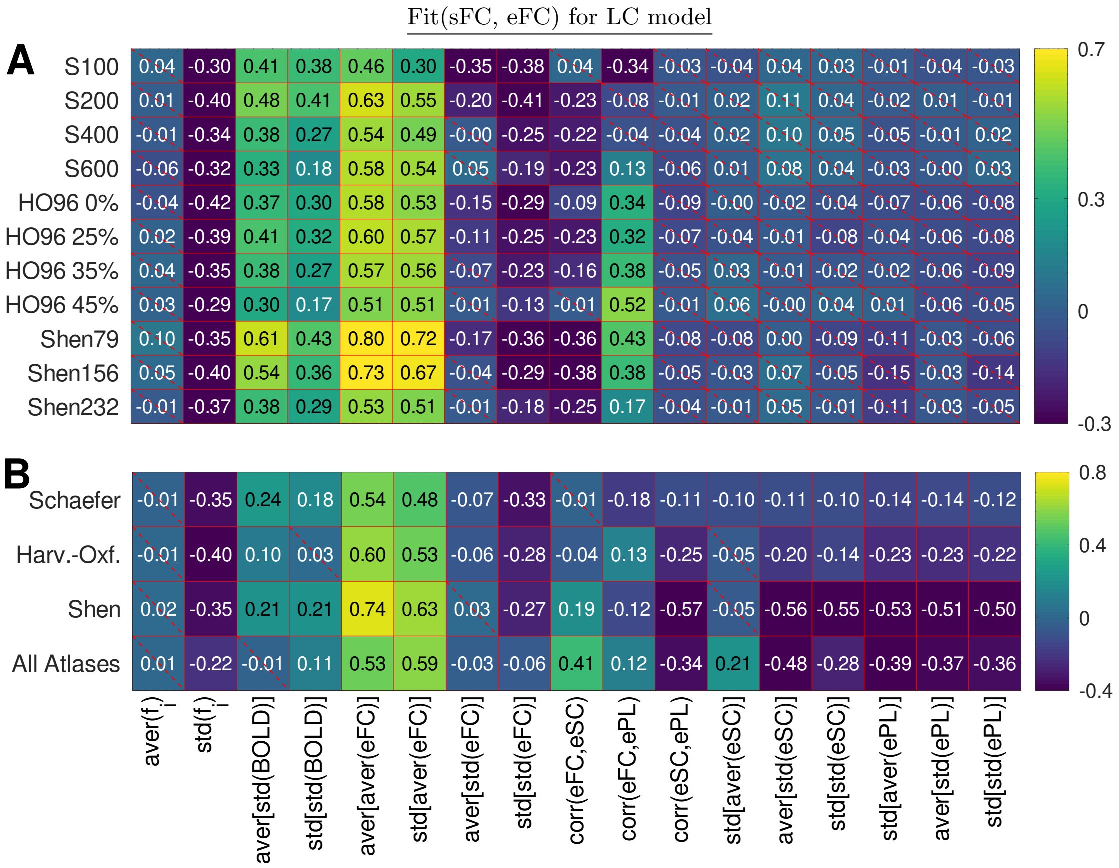

Figure A.4: Relationship between the fitting results (3) of LC model (2) and empirical data. (**A, B**) Pearson correlation across individual subjects between the maximal similarity Fit(sFC, eFC) and several data variables indicated on the horizontal axis. The correlation was calculated for (**A**) different individual parcellations indicated on the vertical axis and (**B**) joint data merged over a few combinations of the considered parcellations as indicated on the vertical axis: all parcellations of the Schaefer atlas, Harvard-Oxford atlas, Shen atlas and all 11 considered parcellations (last row). The correlation is depicted by color, and its magnitude is indicated in the plot. The crossed out cells indicate that the corresponding correlation does not reach the statistical significance with $p < 0.05$.

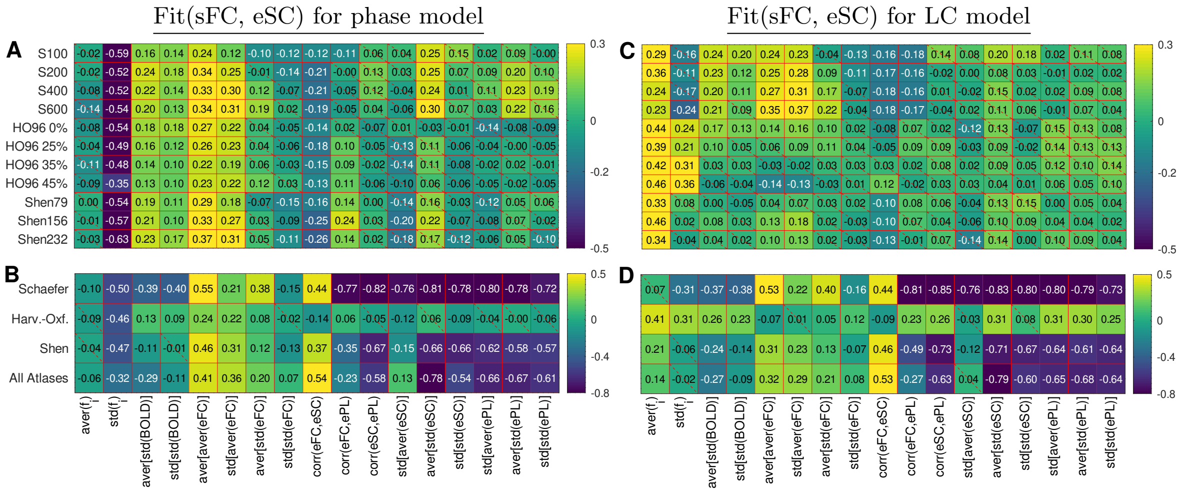

Figure A.5: Relationships between the results of the structure-functional model fitting and empirical data. Pearson correlation between the maximal similarity Fit(sFC, eSC) and several statistical properties (data variables) extracted from the empirical data (see text for details) indicated on the horizontal axes for different parcellations and their combinations (vertical axes). The correlation was calculated for (**A, B**) phase model (1) and (**C, D**) LC model (2), and for (**A, C**) different individual parcellations and (**B, D**) joint data merged over a few combinations of the considered parcellations (vertical axes): all parcellations of the Schaefer atlas, Harvard-Oxford atlas, Shen atlas and all 11 considered parcellations (last row). The correlation is depicted by color, and its magnitude is indicated in the plot. The crossed cells indicate that the corresponding correlation does not reach the statistical significance with $p < 0.05$.

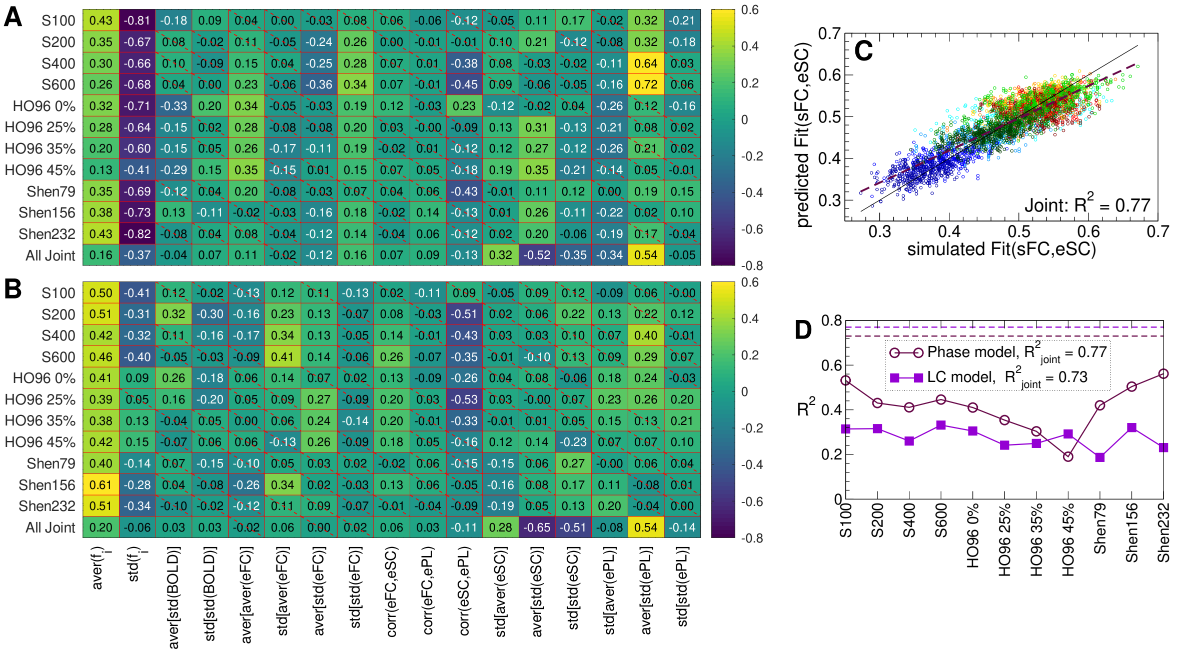

Figure A.6: Modeling the maximal structure-functional similarity Fit(sFC, eSC) by the multiple linear regression (MLR) model with data variables from Fig. 7 as independent variables. (**A, B**) Regression coefficients of the MLR model of Fit(sFC, eSC) for all considered parcellations including the joint data as indicated on the vertical axes for (**A**) phase model (1) and (**B**) LC model (2). The values are depicted by color, and they magnitudes are shown in the plots. The crossed out cells indicate that the corresponding coefficient does not reach the statistical significance with $p < 0.05$. (**C**) Scatter plot for joint data merged over all considered parcellations with the regression line of the Fit-values predicted by MLR versus Fit(sFC, eFC) obtained by simulations of the phase model (1). The diagonal is depicted by thin black line for comparison. The fractions of the explained variance R^2 is also shown in the scatter plot and in plot (**E**) for all individual parcellations for both phase and LC models as indicated in the legend. The dashed lines depict R^2 for the joint data also indicated in the legend.
